# Multi‐Force‐Driven Self‐Recoverable SWIR Mechanoluminescence for Underwater Communication

**DOI:** 10.1002/advs.202523643

**Published:** 2026-01-20

**Authors:** Jie Sun, Yingqiang Li, Lei Wang, Li Li, Bo Zhao, Yu Wang, Yu Zhang, Xian Zheng, Jiale Zhang, Xinhong Wen, Guodong Zhang, Zhijun Wang, Panlai Li, Hao Suo

**Affiliations:** ^1^ National‐Local Joint Engineering Laboratory of New Energy Photoelectric Devices Hebei Key Laboratory of Optic‐electronic Information and Materials College of Physics Science & Technology Hebei University Baoding China

**Keywords:** doping, mechanoluminescence, phosphor, short‐wavelength infrared

## Abstract

Mechanoluminescence (ML) materials featuring photon emission by mechanical stimuli deliver sustainable solutions for frontier applications such as underwater communication. However, the practical utilization of current ML systems has been largely constrained by single‐mode force responsiveness, low cyclic repeatability, and weak resistance to environmental interferences. Herein, we report a new class of MgNb_2_O_6_:Cr^3+^ crystals for the self‐recoverable generation of short‐wavelength infrared (SWIR) emission across multiple forms of mechanical action. Mechanistic investigations affirm the synergistic contribution of piezoelectric and triboelectric effects, thereby yielding high‐brightness and cyclic‐repeatable SWIR‐ML under long‐term mechanical loads. Strikingly, the coupling effect enables unprecedented stable ML signals over 4 000 continuous stretching cycles, which is hitherto inaccessible in the SWIR range. Our findings provide rational design guidelines for multimodal‐force‐driven self‐recoverable and cyclically stable ML material systems, which raise new possibilities for reliable underwater communication technologies.

## Graphical abstract

This study represents the first demonstration of self‐recoverable mechanoluminescent (ML) within a single material across diverse mechanical modes, enabled by the synergistic coupling of piezoelectric and triboelectric effects. Through a combinatorial doping approach, the MgNb_2_O_6_ crystal exhibits breakthrough short‐wavelength infrared ML performance. Notably, the composite elastomer delivers stable ML over 4 000 stretching cycles, thereby enabling reliable underwater communication.

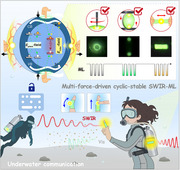

## 1 Introduction

Mechanoluminescence (ML) materials, capable of sustainably emitting light in response to various types of mechanical excitation like compression, stretching, and friction, have risen as hotspot materials with broad technological importance [1, 2, 3]. This unique mechano‐to‐photon conversion principle has enabled tremendous opportunities in a wide spectrum of forefront applications such as intelligent sensing and energy harvesting devices [4, 5, 6, 7, 8, 9]. In particular, ML‐based lighting technologies have shown great promise for underwater wireless exploration, as their self‐powered light emission driven by uninterrupted environmental energy outperforms conventional illumination devices in power consumption, durability, and stretchability under extreme ocean conditions [10, 11]. Nevertheless, optical transmission in real‐world turbid water is severely constrained by absorption and scattering effects, underscoring the critical demand for water‐stable and high‐brightness ML systems with reduced underwater signal losses [12].

**FIGURE 1:**
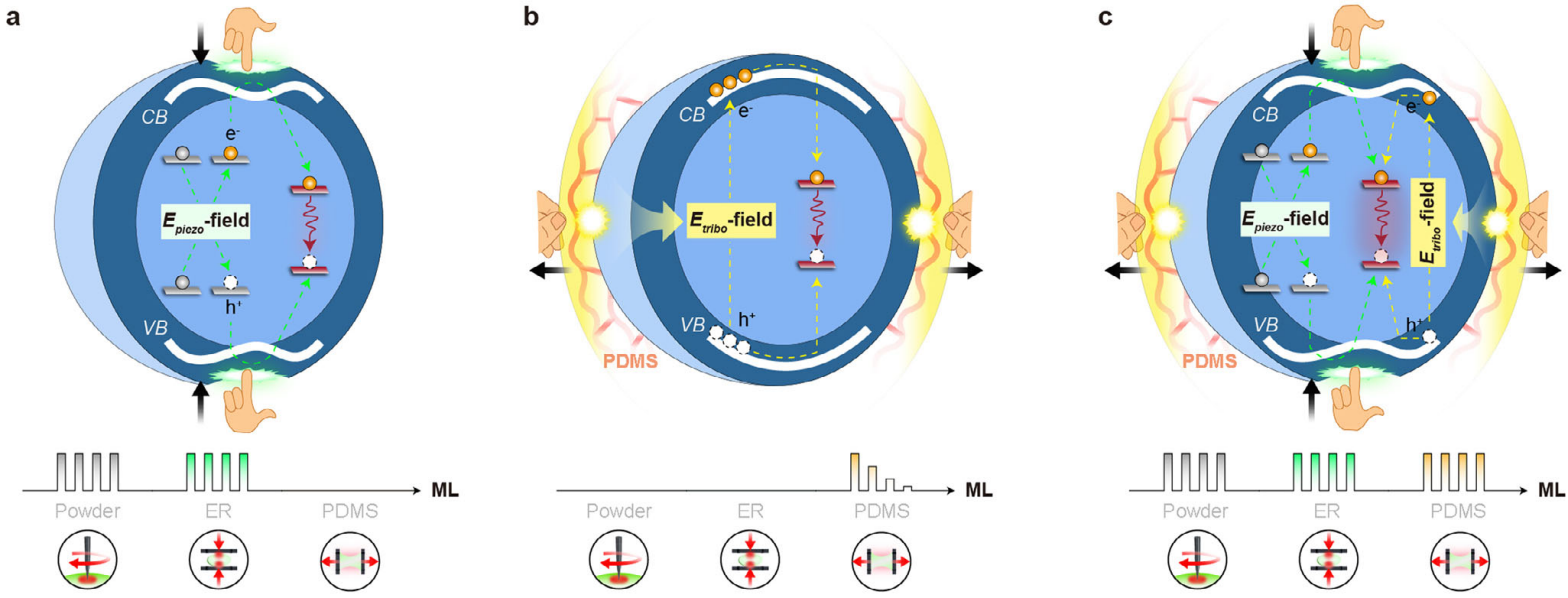
Schematic illustration of mainstream self‐recoverable ML mechanisms. a) The piezoelectric field (*E*_piezo_)‐driven model can render photon emissions through elastic deformation of the materials under mechanical action, allowing self‐recoverable ML in fine‐powder form under frictional loads or in a rigid ER substrate under compressive stress. b) The triboelectric field (*E*_tribo_)‐induced model can deliver pre‐irradiation‐free ML in inorganic–organic composite elastomers through interfacial displacement between two phases under mechanical action. However, most existing systems suffer from poor ML repeatability when subjected to stretching, while isolated powders hardly respond to all forms of external forces. c) The proposed model exploits the synergistic coupling of piezoelectric and triboelectric effects, thereby enabling high‐brightness and cyclically stable ML of MgNb_2_O_6_:Cr^3+^ whether in powder, ER, or PDMS composite forms, across diverse forms of mechanical actions.

Recent investigations into ML materials have significantly expanded the physical mechanism framework from the energy‐prestored model to a self‐recovered one, which avoids photoexcitation and renders essentially sustainable light emissions under sole mechanical action [13, 14]. Typically, this self‐recoverable ML process is initiated via the internal or external electric field generated upon direct force excitation [15]. For example, compression‐induced repeatable ML has been found in several piezoelectric materials (e.g., Ca(Sr)ZnOS and LiTaO_3_) in powder form or rigid substrates (i.e., epoxy resin, ER), likely driven by a local piezoelectric field (Figure 1a) [16, 17]. When embedded into flexible polydimethylsiloxane (PDMS), certain non‐piezoelectric crystals (e.g., Lu_3_Al_5_O_12_:Ce^3+^ and CaF_2_:Tb^3+^) can also produce self‐recoverable ML upon friction via interfacial triboelectrification (Figure 1b) [18, 19].

Until now, the self‐recoverability and cyclic repeatability of existing ML materials have still been confined to a single‐mode mechanical excitation (stress → piezoelectric or friction → triboelectric), greatly hampering their practical applicability in multi‐modal stress‐field scenarios (Table S1) [20, 21]. More critically, only a few ML elastomers have demonstrated excellent cyclic stability under rapid stretching mode over 1 000 cycles, represented by ZnS:Cu@PDMS [22, 23, 24]. On a side note, the self‐recoverable ML is primarily available in the visible and near‐infrared region, which suffers from much stronger radiation interference and scattering effects in complex environmental conditions (e.g., haze, fog, dust, or turbid water) compared to the short‐wavelength infrared (SWIR, 900–1700 nm) range [25, 26]. Therefore, achieving self‐recoverable SWIR‐ML with high cyclic stability in response to diverse mechanical modes is highly valuable to advance practical applications, yet remains an unfulfilled research to date.

In this study, we establish a design principle to enable self‐recoverable ML within a single host across multiple forms of mechanical action, based on the synergistic coupling of piezoelectric and triboelectric effects (Figure 1c). We show that MgNb_2_O_6_:Cr^3+^ crystal, whether in powder, rigid, or flexible polymer forms, consistently delivers high‐brightness broadband SWIR‐ML under long‐term compressive and frictional loads. More importantly, we realize, for the first time, highly repeatable and cyclically stable SWIR‐ML output over thousands of stretching modes. By leveraging this unprecedentedly durable and water‐stable SWIR‐ML, we further developed a self‐powered wearable display for encrypted communication and safety surveillance in harsh underwater environments.

## 2 Results and Discussion

### 2.1 Structural and Optical Characterization

**FIGURE 2:**
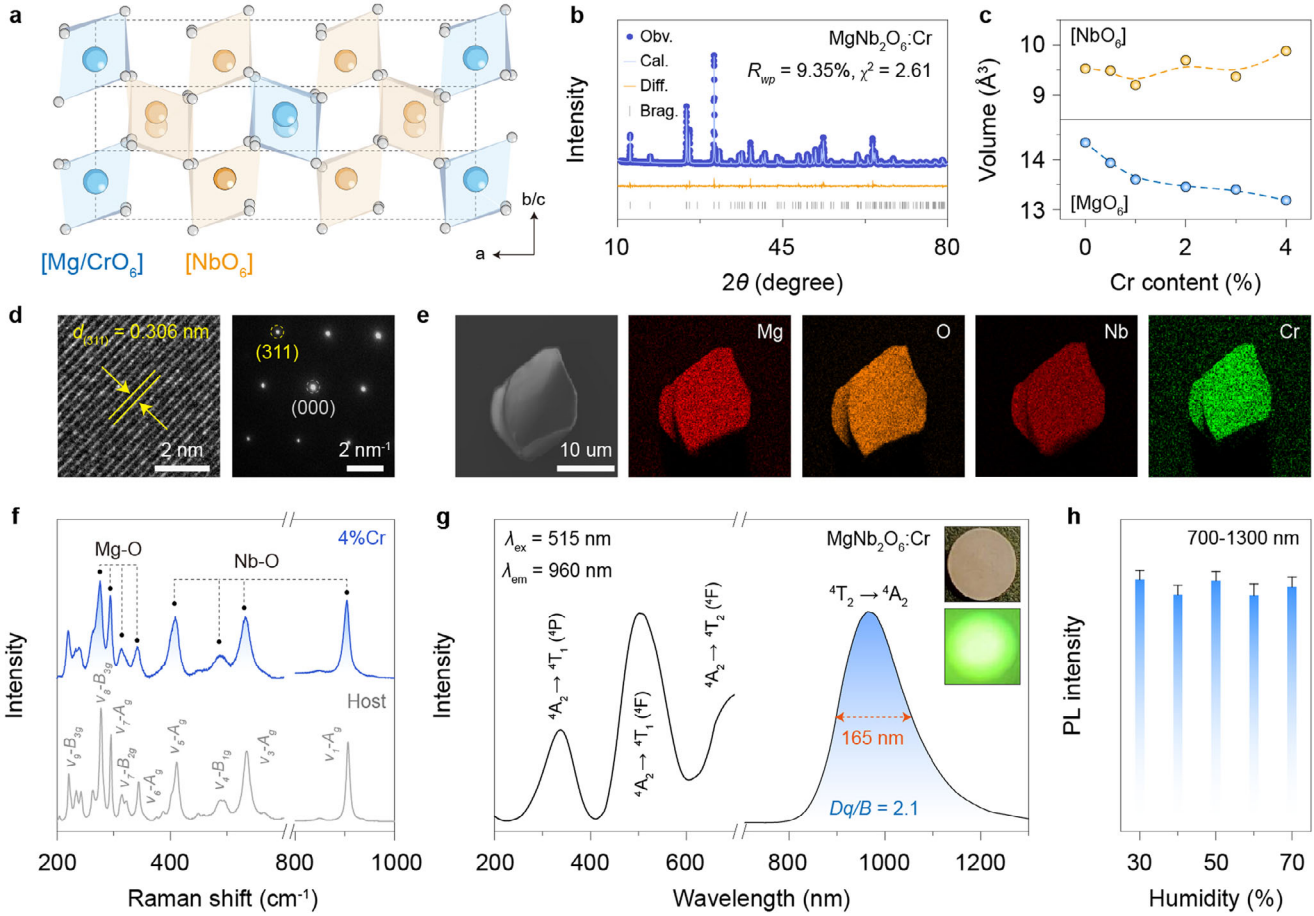
Structural characterization and PL property of MgNb_2_O_6_:Cr^3+^. a) Schematic presentation of MgNb_2_O_6_ crystal structure highlighting [MgO_6_] and [NbO_6_] units. b) Rietveld refinement of XRD pattern for Cr^3+^‐doped MgNb_2_O_6_ crystals. c) Rietveld refinement results of [NbO_6_] and [MgO_6_] volumes as a function of Cr^3+^ content. d) HR‐TEM and corresponding SAED pattern of a randomly selected microcrystal. Note that the obtained interplanar spacing of 0.306 nm can be indexed as the (311) plane of orthorhombic MgNb_2_O_6_. e) EDX elemental mapping of a single MgNb_2_O_6_:Cr^3+^ (2%) microparticle. f) Raman spectra of bare and Cr^3+^‐doped MgNb_2_O_6_ crystals. Note that the main peaks at 220.5, 277.3, 295.1, 313.9, 343.7, 411.5, 490.9, 535.1, 906.8 cm^−1^ can be assigned to *υ_9_‐B_3g_*, *υ_8_‐B_3g_*, *υ_7_‐A_g_*, *υ_7_‐B_2g_*, *υ_6_‐A_g_*, *υ_5_‐A_g_*, *υ_3_‐A_g_*, and *υ_1_‐A_g_* modes, respectively. [32] g) PLE (*λ*_ex_ = 960 nm) and PL (*λ*_ex_ = 515 nm) spectra of MgNb_2_O_6_:Cr^3+^ (2%), and insets show the corresponding photographs under daylight or 275 nm UV light recorded by a night‐vision monocular. h) Integral PL intensity (700–1300 nm) as a function of ambient relative humidity (RH ∼ 30–70%). Note that the humidity around the sample was approximately controlled using a humidifier, and the error bars of emission intensity represent the standard deviations from three sets of repeated measurements.

Our study employs trivalent chromium ion (Cr^3+^, [Ar]3d^3^) as a model dopant capable of producing tunable broadband ML emissions across the NIR and SWIR range [27, 28]. The columbite‐like magnesium niobate (MgNb_2_O_6_) is chosen as the ML host because of its high physicochemical stability, wide transparent range, and excellent electro‐optical characteristics, which crystallizes in an orthorhombic structure (space group: *Pbcn*) with *D_2h_* symmetry [29]. Specifically, distorted octahedral [MgO_6_]/[NbO_6_] units form independent edge‐sharing zig–zag clusters that are corner‐connected to establish an ordered layered structure (Figure 2a). This octahedral network provides an ideal crystal‐field environment for Cr^3+^ incorporation, which also offers the potential to create local piezoelectricity for efficient ML through inversion‐symmetry breaking [30].

Through a high‐temperature solid‐state reaction, the resulting pink powders were confirmed, by X‐ray powder diffraction (XRD) measurement, to be a pure orthorhombic phase of MgNb_2_O_6_ (Figure 2b). Notably, we observed a continuous shift of diffraction peaks toward larger angles, suggesting the successful occupation of Cr^3+^ (0.615 Å, CN6) at larger octahedral sites (Figure S1). This statement was validated by a steady shrinkage of unit cell and [MgO_6_] octahedron upon Cr^3+^ doping based on the Rietveld refinement analysis (Figure 2c; Figure S2 and Table S2). The slight alteration in [NbO_6_] volume also supports that Cr^3+^ preferentially occupies the Mg^2+^ rather than Nb^5+^ site, due to their closer valence state and stronger ligand‐field stabilization energy. This site occupation scheme is further corroborated by the density functional theory (DFT) calculations, which showed a lower formation energy (*E_form_*) for Cr^3+^ in the [MgO_6_] compared to the [NbO_6_] site (Table S3). In addition, the high‐resolution transmission electron microscopy (HR‐TEM) and associated selected area electron diffraction (SAED) results demonstrate the remarkable crystalline nature of the orthorhombic crystal (Figure 2d).

Energy‐dispersive X‐ray analysis (EDX) reveals the uniform distribution of the host and dopant elements across a single microparticle (Figure 2e). Such compositional homogeneity is likely beneficial for self‐recoverable ML with stable outputs, as it enables efficient and homogeneous stress‐induced piezoelectric effect under mechanical stimulation. The successful incorporation of trivalent chromium was further confirmed by the appearance of two distinct peaks at 576.1 and 585.5 eV in X‐ray photoelectron spectroscopy (XPS), attributed to 2p_3/2_ and 2p_1/2_ of Cr^3+^ (Figure S3). Raman spectroscopy was employed to identify the octahedral phonon energies, which clearly show main peaks in the 200–350 and 400–1000 cm^−1^ ranges corresponding to the vibration modes of [MgO_6_] and [NbO_6_], respectively (Figure 2f). Notably, the linewidth of Raman peaks broadened significantly upon Cr^3+^ doping, indicating a doping‐mediated distortion of the local symmetry (Figure S4) [31].

We next studied the photoluminescence (PL) behavior of the Cr^3+^‐doped MgNb_2_O_6_ crystal. Upon excitation at 515 nm, the emission spectrum consists of an intense single broadband centered at 960 nm in the SWIR range due to the spin‐allowed ^4^T_2_ → ^4^A_2_ transition of the Cr^3+^ dopant, yielding a large full width at half maximum (FWHM) of 164 nm (Figure 2g). This observation indicated that Cr^3+^ was subjected to a weak crystal‐field strength, as evidenced by the small crystal‐field parameter (*Dq/B* ∼ 2.1) derived from the absorption peak energy (Figure S5). Notably, this long‐wavelength broadband emission featured high resistance to ambient humidity, owing to the exceptional stability of the oxide host (Figure 2h).

### 2.2 Self‐Recoverable SWIR‐ML Performance

**FIGURE 3:**
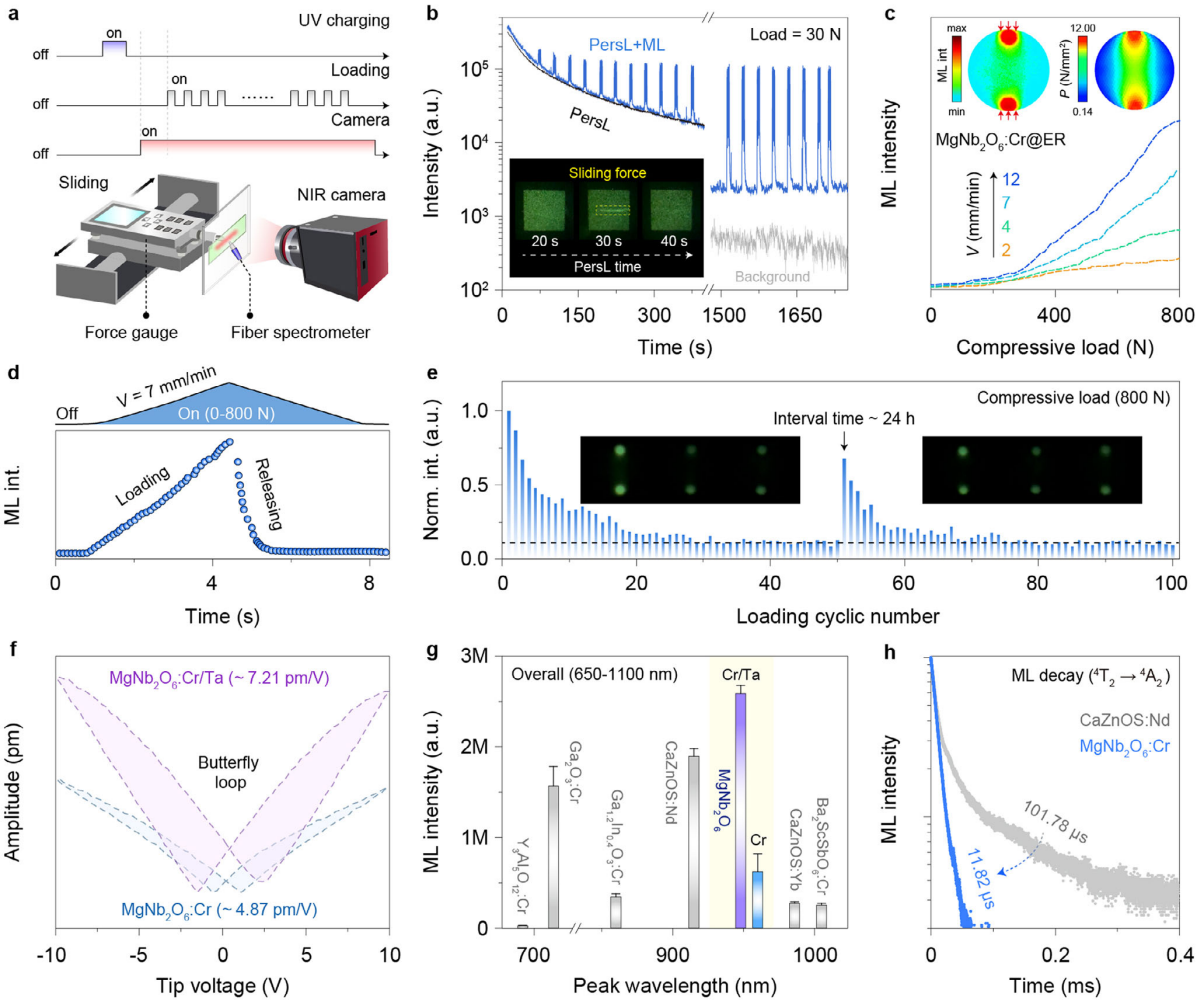
Self‐recoverability and mechanism of ML powders and hard pellets. a) Schematic of the homemade apparatus for ML characterization. A control unit comprising a linear motor and a force gauge was employed to apply quantitative mechanical excitation. The optical signal (ML or PersL) was analyzed by a night‐vision monocular (intensity) and a fiber‐coupled spectrometer (spectrum). b) PersL decay curves of MgNb_2_O_6_:Cr^3+^ (2%) with and without continuous pulse force excitation (30 N, 0.033 Hz) after 30 s of pre‐charging at 254 nm, and insets show photographs of the sample under single‐sliding action during the PersL process. Note that the intensity was calculated by summing up the grayscale values of the pixels within each recorded NIR image. c) ML intensity of MgNb_2_O_6_:Cr^3+^@ER pellet as a function of applied compressive load at varied loading velocities (2–12 mm/min). Insets show the spatial distribution of relative ML intensity, along with the simulated stress distribution of the pellet based on the finite element analysis. d) Time‐resolved ML intensity curve during a compression‐release cycle (bottom), along with the corresponding applied force variation at a fixed loading velocity of 7 mm/min (top). e) The ML recoverability and cyclic stability of the hard pellet under repeated compressive loads of 800 N, with insets showing the ML images. The partial ML recovery of the pellet after a 24‐hour rest period indicated that the initial intensity loss was due to structural degradation rather than poor self‐recoverability. f) PFM amplitude of MgNb_2_O_6_:Cr^3+^ without and with Ta^5+^ co‐doping under a tip bias in the range of  ± 10 V. The observed amplitude butterfly loop indicated the presence of piezoelectric effect. g) Comparsion of integral ML intensity among as‐prepared crystals and the reported self‐recoverable NIR‐ML materials under force excitation at 50 N. Note that these composite films were fabricated and tested under strictly identical experimental conditions. h) ML decay curves of MgNb_2_O_6_:Cr^3+^ and CaZnOS:Nd^3+^ under impact excitation. A free‐falling ball is applied to generate impaction on the ML film, and the ML signals are collected by a photomultiplier tube.

Upon continuous grinding, fine‐powder MgNb_2_O_6_:Cr^3+^ displayed bright self‐recoverable ML without the need for pre‐charging, easily detectable by a night‐vision monocular (Video S1). For quantitative ML analysis, we assembled a specialized measurement system that comprises a customized mechanical control unit, an NIR camera, and a fiber‐coupled charge‐coupled device (CCD) spectrometer (Figure 3a). The powder sample was encapsulated between transparent polyethylene terephthalate (PET) sheets for measurements. Under mechanical loading of 15 N, the acquired ML spectrum closely resembled the PL counterpart, validating the pivotal involvement of Cr^3+^ transition in the ML process (Figure 3b; Figure S6a). In line with PL observations, the optimal Cr^3+^ concentration was found to be 2% with the highest ML intensity (Figure S6b). Notably, the sample showed a nearly linear ML enhancement with increasing applied loads, highlighting the promise for stress sensing applications (Figure S6c).

The self‐recoverable nature of SWIR‐ML was substantiated by the nearly constant ML intensity under continuous force excitation (Figure S7a). This external force had minimal effect on the morphology and grain size of the original microparticles, ruling out the fracture‐induced ML as the dominant mechanism (Figure S7b). On a separate note, the trap‐mediated model fails to explain the ML behavior in MgNb_2_O_6_:Cr^3+^ crystal, as evidenced by the unaltered ML intensity after depleting the energy stored in trap states via thermal bleaching pre‐treatment at various temperatures (Figure S8a). This assumption was further supported by the mechanistic independence between the persistent luminescence (PersL) and ML processes. Specifically, repeated force stimulation elicited a transient photon enhancement via the ML process, without accelerating the PersL decay after UV charging (Figure 3b). Consistently, in situ thermoluminescence (TL) spectra remained essentially unchanged before and after force stimulation, suggesting that ML photons are unlikely to originate from the PersL‐involved trap states (Figure S8b).

The mechano‐to‐optical responsiveness of ML emitters was further examined under compressive load to elucidate the underlying ML mechanism. The powder sample was embedded in rigid ER to ensure efficient stress transfer and structural integrity with suppressed inter‐particle displacement. Remarkably, the ML brightness scaled linearly with the compressive loads, whereas higher loading velocities led to enhanced force sensitivity (Figure 3c; Figure S9) [17]. The finite element analysis corroborated the direct visualization of stress distribution by the SWIR‐ML across the compressed pellet. However, a marked contrast was observed upon stress release, as the ML diminished rapidly before complete unloading (Figure 3d). Additionally, bright and self‐recoverable ML signals were consistently captured over consecutive compression‐release cycles (Figure 3e; Figure S10 and Video S2). The initial decline in ML intensity was likely attributed to structural deformation of the hard composites during early compressive strain, followed by gradual stabilization that elicited a steady ML output [17].

We thus deduce that the self‐recoverable ML may stem from the doping‐induced local piezoelectricity, as supported by the piezoresponse force microscopy (PFM). Specifically, the presence of characteristic butterfly loops in MgNb_2_O_6_:Cr^3+^ confirmed its pronounced piezoelectric behavior (*d_33_* ^*^ = 4.87 pm/V, Figure 3f; Figure S11). Note that the piezoelectric effect was barely affected by the Cr^3+^ concentration at a low doping level (Figure S12). Moreover, our DFT calculations identify additional 3d states near the conduction band minimum upon Cr^3+^ doping (Figure S13). This doping‐induced perturbation of inversion symmetry likely gives rise to a localized piezoelectric field under mechanical stress, accompanied by energy band tilting. Driven by the inner piezo‐potential, charge carriers are released and separated from neutral defect centers, followed by radiative recombination through the Cr^3+^ levels to produce broadband SWIR‐ML (Figure S14) [33, 34].

Further investigation into the role of piezoelectricity in the ML process was conducted using Ta^5+^ ion as an exemplary co‐dopant. The comparable ionic radius of Ta^5+^ to Nb^5+^ (0.64 Å) enabled the formation of homogeneous solid solutions without inducing noticeable spectral shifts (Figure S15). To our delight, this targeted substitution markedly amplified the piezoelectric response (*d_33_* ^*^ = 4.87 → 7.21 pm/V, Figure 3f), leading to a 4.2‐fold enhancement of broadband SWIR‐ML (Figure 3g; Figure S16) [30]. It is worth noting that the ML improvement is not ascribed to Ta^5+^‐induced defect engineering, due to the independent nature of ML and PersL (Figure S17). Accordingly, the pronounced piezoelectric effect gives rise to strong ML comparable to benchmark NIR‐ML material systems (Figure S18). This exceptional performance is rarely achievable in the long‐wavelength region beyond 900 nm due to the inherently large Stokes shift [26, 35]. As an added benefit, the d‐d transition rendered a rapid ML decay on the microsecond scale (∼ 11.82 µs) under impact loading, over one order‐of‐magnitude faster than that of lanthanide emitters (e.g., CaZnOS:Nd^3+^ ∼ 101.78 µs), thereby affording exceptional temporal resolution for high‐frequency dynamic stress sensing (Figure 3h; Figure S19) [36].

**FIGURE 4:**
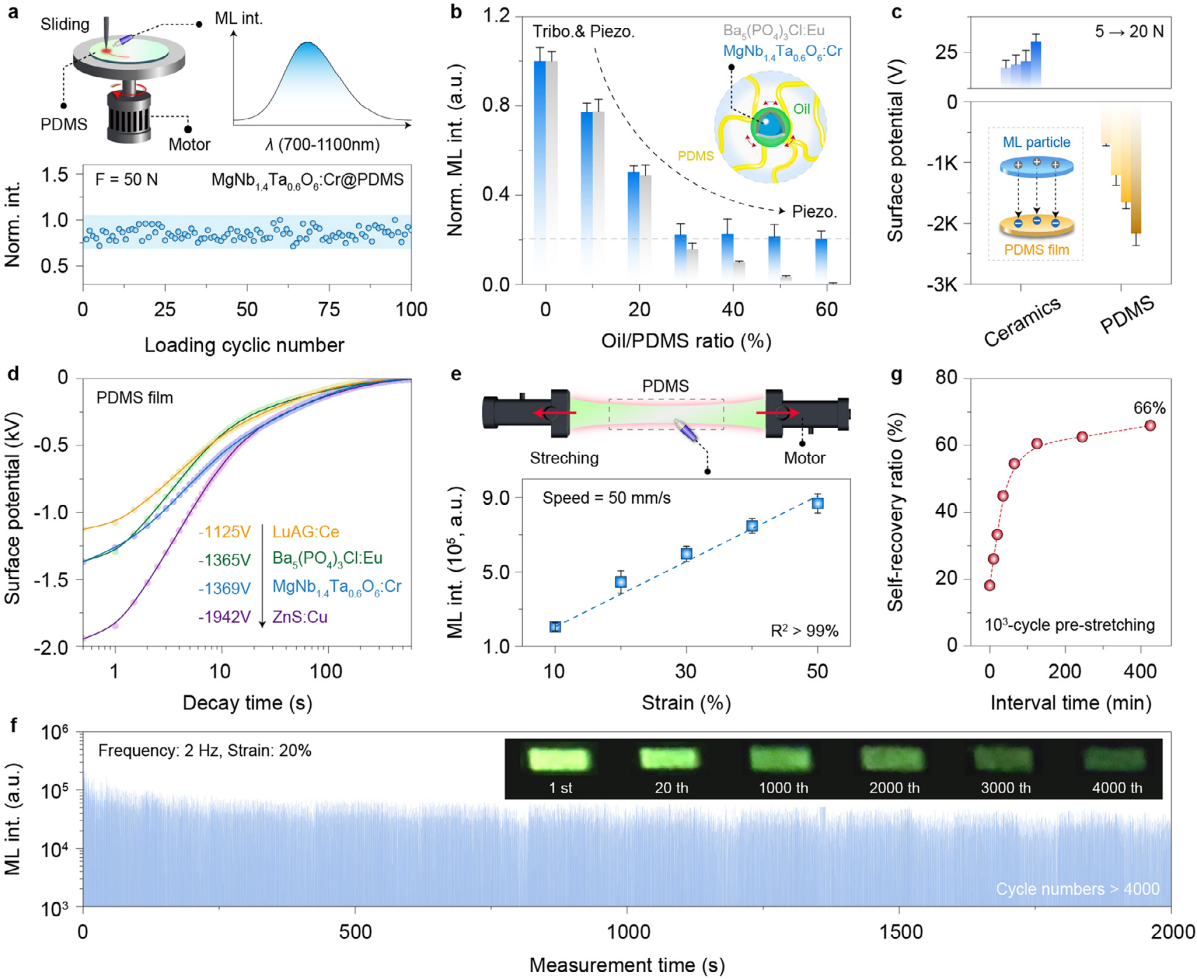
Self‐recoverability and mechanism of flexible ML elastomers. a) Schematic of the homemade apparatus for ML measurement under rotational sliding excitation, along with the recorded ML spectrum and cyclic stability of MgNb_1.4_Ta_0.6_O_6_:Cr^3+^@PDMS composites. b) The variation of normalized integral ML intensity for MgNb_1.4_Ta_0.6_O_6_:Cr^3+^@PDMS as a function of lubricant oil concentration, along with the well‐established triboelectric ML material Ba_5_(PO_4_)_3_Cl:Eu^2+^@PDMS as the control.^[^ ^38^ ^]^ Note that the residual ML intensity after adding lubricant oil is likely attributed to the piezoelectric effect, as the control group showed no triboelectric ML under the same conditions. c) Triboelectric potential of the ML ceramic and PDMS film following 1 min of reciprocating friction under different loads, and the inset illustrates the possible electron transfer process. d) Decay curves of triboelectric potential on the PDMS surface after friction (F = 10 N) with different ML ceramics. The initial potential values were ‐1125, ‐1365, ‐1369, and ‐1942 V for Lu_3_Al_5_O_12_:Ce^3+^, Ba_5_(PO_4_)_3_Cl:Eu^2+^, MgNb_1.4_Ta_0.6_O_6_:Cr^3+^, and ZnS:Cu, respectively. e) Schematic of the universal test machine for ML measurement under stretching excitation, along with the recorded ML intensity under different tensile strains. f) The ML recoverability and stability of the composite elastomer over 4,000 stretching cycles, with insets showing the corresponding ML images at different stretching cycles. g) Self‐recovery of ML intensity relative to the initial value after the pre‐stretched film (∼ 1000 cycles) was placed at room temperature for different durations.

Beyond the piezoelectric effect in powders or hard substrates, we conducted an extended evaluation of ML performance in flexible elastomers, which is essential for intelligent wearable devices [22]. When embedded in high‐elasticity PDMS polymer, the sample emitted bright and self‐recoverable SWIR‐ML under continuous rotational scratching, without requiring any external charge supply (Figure 4a). Since scratching action involves both compressive and frictional loads, dimethyl silicone oil was intentionally added as the lubricant to suppress the friction coefficient between inorganic particles and organic elastomers, leading to a marked attenuation of ML intensity (Figure 4b; Figure S20) [37]. Note that the residual ML originates from the stress‐induced piezoelectric effect, as evidenced by the detectable ML on the inner side of the elastomer under bending deformation (Figure S21). These observations established that the ML in flexible elastomers arises from the synergistic interplay between the piezoelectric effect (compression‐driven) and the interfacial triboelectric effect (friction‐driven).

A distinct feature of the triboelectricity‐induced ML lies in its strong dependence on the triboelectric charge between the inorganic and organic phases [21, 38]. Accordingly, we quantitatively assessed the surface triboelectric potential using an electrostatic sensor after cyclic rubbing between a compact sample of ceramics and a pure organic film. The measured surface potentials confirmed that interfacial triboelectricity was generated by direct electron transfer from the sample to PDMS during frictional contact (Figure 4c). Under this triboelectric field, the electrons gained by PDMS are expected to back‐transfer and bombard the luminescence centers, in a manner analogous to the cathodoluminescence (CL, Figure S22) [39]. Notably, the MgNb_1.4_Ta_0.6_O_6_:Cr^3+^@PDMS composite delivered the strongest SWIR‐ML, attributed to the highest negative charge affinity of PDMS compared with other organic phases (i.e., polyurethane and silicone; Figure S23). The obtained relative surface potential (∼ −1369 V) was even comparable to that of benchmark triboelectric ML materials (e.g., ZnS:Cu ∼ −1942 V and Ba_5_(PO_4_)_3_Cl:Eu ∼ −1365 V), thereby producing a strong triboelectric field to activate ML upon friction (Figure 4d; Figure S24). This superior performance was further corroborated by detectable ML even during the releasing process with a much weaker triboelectric effect (Figure S25).

In a further set of experiments, we captured the intense SWIR‐ML of flexible elastomer under rapid elastic stretching, which is the dominant deformation mode imposed on wearable devices during repetitive large‐area strain from body motion (Video S3). In line with the surface potential variations, the ML intensity was positively correlated with the tensile strain and stretching speed, reinforcing the essential role of interfacial triboelectricity (Figure 4e; Figure S26). Synergistically coupled with the piezoelectric field, this strong triboelectric field enabled unprecedented repeatable and cyclically stable SWIR‐ML output over 4 000 stretching‐releasing actions (Figure 4f). It is worth noting that the ML intensity showed a sharp decrease at the onset of stretching, which may be due to the partial break of the relatively weak hydrogen bonds and Van der Waals’ force at the interface between the ML particles and the PDMS chains. Since the interfacial interactions possess a certain degree of reversibility and self‐healing capability under ambient conditions, the degraded ML intensity can be partially restored after a resting period of the stretched elastomer (Figure 4g) [24].

### 2.3 Self‐Powered SWIR‐ML for Underwater Communication

**FIGURE 5:**
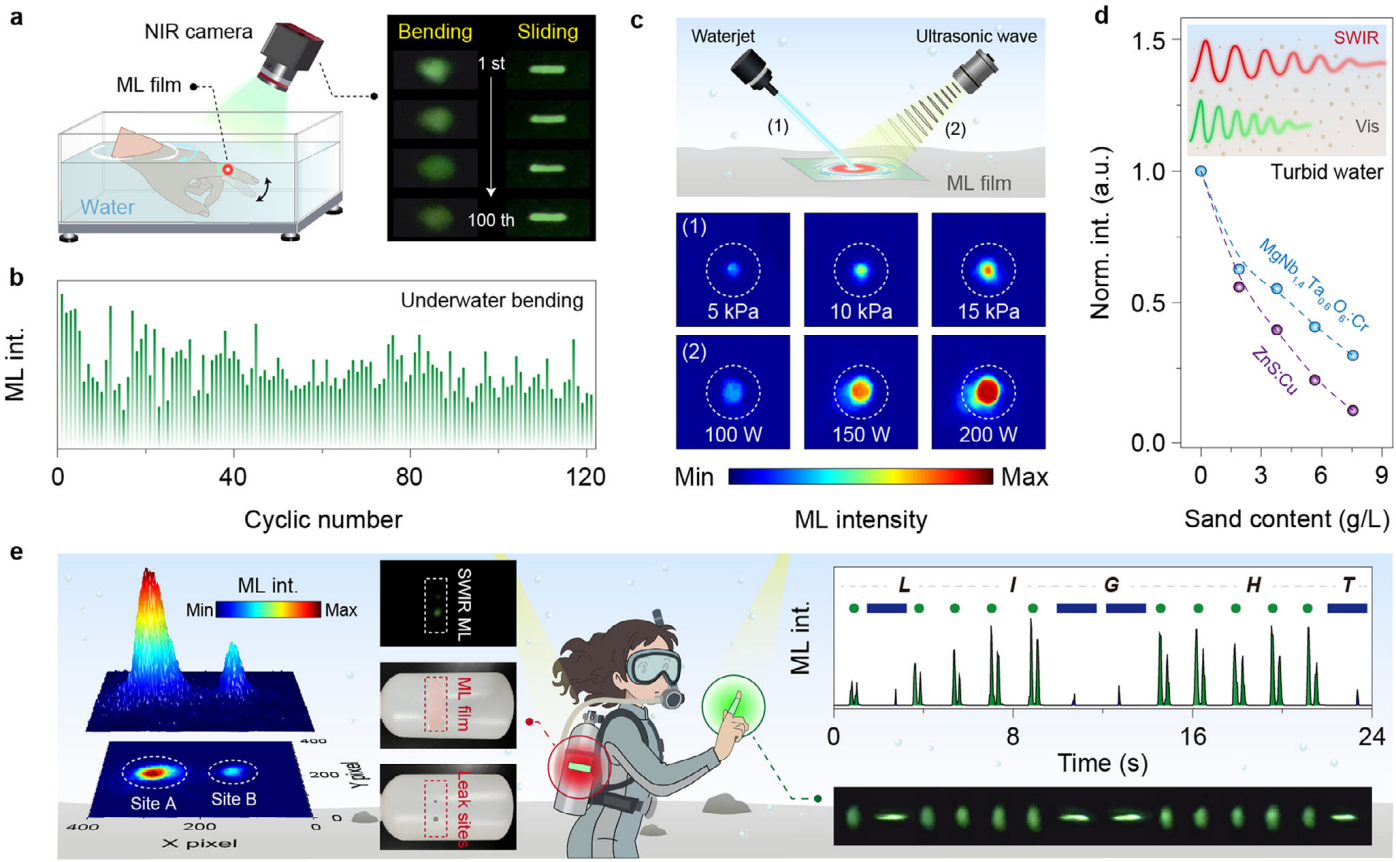
Self‐powered SWIR‐ML display for underwater communication. a) Schematic illustration of the experimental setup for underwater ML measurements, along with recorded ML images of the MgNb_1.4_Ta_0.6_O_6_:Cr^3+^@PDMS film at different bending and scratching cycles. The film was tightly adhered to the forefinger surface, and the ML signal during the finger movement was captured using a night‐vision monocular positioned above the waterline. b) The cyclic stability of underwater ML intensity under repeated bending actions over 100 cycles. c) Schematic illustration of the SWIR‐ML driven by dynamic water energy from a waterjet or an ultrasonic cabinet, along with corresponding ML images at various water pressures (∼ 5–15 kPa) or ultrasonic powers (∼ 100–200 W). d) The light losses after passage through turbid water for MgNb_1.4_Ta_0.6_O_6_:Cr^3+^@PDMS and ZnS:Cu@PDMS films, with the inset showing the schematic dependence of penetration capability on light wavelength. The water turbidity was adjusted by adding suspended fine‐sand particles to the aqueous solution, and the PL signal was measured under 275 nm excitation for better evaluation. e) Conceptual diagram of underwater applications using a self‐powered wearable SWIR‐ML skin. The left section illustrates leakage detection of a simulated gas tank with the ML film attached, showing the simulated gas tank with two leakage sites and the corresponding spatial ML distribution. The right section depicts the generation of dot (·) and dash (‐) in Morse code by joint bending and fingertip scratching, respectively, with real‐time ML outputs and images during the encrypted transmission of the message “LIGHT”.

Building on the exceptional stability of the oxide host, we further assessed the SWIR‐ML performance in underwater settings (Figure 5a). After immersion in aqueous solution, the composite elastomer largely preserved its highly reproducible ML behavior under scratching or stretching actions (Figure 5b; Figure S27). Notably, the SWIR‐ML is highly resistant to a series of polar solvents even after prolonged soaking over several months (Figure S28). It is worth mentioning that the slight ML degradation stems from the reduced triboelectric effect due to the inevitable water infiltration into microscopic voids of hydrophobic PDMS film [40]. In addition, variations in temperature and ionic strength led to only a limited attenuation of underwater ML performance (Figure S29). Dynamic mechanical energy from water flow or waves, which is an uninterrupted and abundant energy source in nature, can also be harvested to induce bright and stable SWIR‐ML output (Figure 5c). This dual‐functionality of energy harvesting and ML holds great potential for various self‐powered applications, exemplifying the efficient concept of “turning waste into treasure” [41, 42, 43]. As an added benefit, the longer wavelength of SWIR light typically experiences less scattering, thereby enabling enhanced propagation relative to visible light through real‐world turbid water containing suspended impurities (Figure 5d) [44].

The availability of SWIR‐ML with high brightness and cyclical stability offers significant opportunities for underwater communication. As a proof‐of‐concept, we devised a self‐powered wearable photonic display system by tightly adhering MgNb_1.4_Ta_0.6_O_6_:Cr^3+^@PDMS to the finger surface, enabling underwater light generation driven by finger movements (Figure 5e). Owing to the multimodal force‐responsive ML feature, encrypted transmission is thus achievable through prescribed photonic patterns under specific actions, offering an unprecedented information loading capacity. For example, spot‐ and line‐shaped ML traces generated by joint bending and fingertip scratching could be resolved as dot (·) and dash (‐) in Morse code, respectively. In this way, the concealed message “LIGHT” could be readily deciphered underwater by a sequence of ML pulses. The invisible SWIR signal offers an extra level of encryption to underwater communication. On another note, the SWIR‐ML film enabled the real‐time detection and precise localization of leakage point on a gas cylinder, underscoring its promise for safety surveillance in harsh underwater settings.

## 3 Discussion

In conclusion, our investigation of MgNb_2_O_6_:Cr^3+^ crystals initiates an efficient paradigm for self‐recoverable SWIR‐ML activated by multiple mechanical modes. Benefiting from the synergistic interplay of the piezoelectric and triboelectric effects, we achieve high‐brightness and cyclically stable ML upon long‐term mechanical excitations, outperforming the existing SWIR‐ML materials. In particular, the optimized elastomer delivered unprecedented stable ML output after 4 000 continuous stretching cycles. By virtue of its superior water stability and minimized scattering, this self‐recoverable SWIR‐ML can be harnessed for self‐powered underwater communication and safety surveillance. These advances not only offer a concise principle for attaining multimodal‐force‐driven self‐recoverable ML, but also significantly broaden the utility of ML materials for advanced photonic applications in harsh settings.

## Conflicts of Interest

The authors declare no conflict of interest.

## Supporting information




**Supporting File 1**: advs73961‐sup‐0001‐SuppMat.docx.

**Supporting File 2**: advs73961‐sup‐0002‐VideoS1.mp4.

**Supporting File 3**: advs73961‐sup‐0003‐VideoS2.mp4.

**Supporting File 4**: advs73961‐sup‐0004‐VideoS3.mp4.

## Data Availability

The data that support the findings of this study are available from the corresponding author upon reasonable request.
