# Supplementary material for: Multi‐Force‐Driven Self‐Recoverable SWIR Mechanoluminescence for Underwater Communication
Source: Adv Sci (Weinh). 2026 Jan 20;13(18):e23643. doi: 10.1002/advs.202523643 (PMC13042814; doi:10.1002/advs.202523643)
Supplement: Supplementary file 1 — Supporting File 1: advs73961‐sup‐0001‐SuppMat.docx. [file ADVS-13-e23643-s004.docx]

Supporting Information

**Multi-force-driven Self-recoverable SWIR Mechanoluminescence for Underwater Communication**

*Jie Sun, Yingqiang Li, Lei Wang, Li Li, Bo Zhao, Yu Wang, Yu Zhang, Xian Zheng, Jiale Zhang, Xinhong Wen, Guodong Zhang, Zhijun Wang, Panlai Li, Hao Suo**

Address correspondence to E-mail: suo@hbu.edu.cn

**Experimental Sections**

***Raw Materials and Reagents:*** Magnesium oxide (MgO, 99.99%), niobium pentoxide (Nb_2_O_5_, 99.99%), tantalum oxide (Ta_2_O_5_, 99.99%), chromium oxide (Cr_2_O_3_, 99.95%), aluminum oxide (Al_2_O_3_, 99.99%), gallium oxide (Ga_2_O_3_, 99.99%), indium oxide (In_2_O_3_, 99.99%), lutetium oxide (Lu_2_O_3_, 99.99%), yttrium oxide (Y_2_O_3_, 99.99%), cerium dioxide (CeO_2_, 99.99%), europium oxide (Eu_2_O_3_, 99.99%), ytterbium oxide (Yb_2_O_3_, 99.99%), neodymium oxide (Nd_2_O_3_, 99.99%), scandium oxide (Sc_2_O_3_, 99.99%), and antimony oxide (Sb_2_O_3_, 99%), barium carbonate (BaCO_3_, 99.95%), calcium carbonate (CaCO_3_, 99.95%), lithium carbonate (Li_2_CO_3_, 99.95%) diammonium hydrogen phosphate [(NH_4_)_2_HPO_4_, 99%], and ammonium chloride (NH_4_Cl, 99%) were purchased from Aladdin Biochemical Technology Co., Ltd. Zinc sulfide (ZnS, 99.99%) was purchased from Sigma-Aldrich Co., Ltd, and ZnS:Cu microparticles (D502CT) were obtained from Shanghai Keyan Phosphor Technology Co., Ltd. Dimethyl silicone oil (AR) was purchased from Sinopharm Chemical Reagent Co,. Ltd. Notably, all chemicals and reagents were used as received without further purification.

***Preparation of doped MgNb_2_O_6_ crystals:*** The columbite-type MgNb_2_O_6_ crystals doped with Cr^3+^ ions were synthesized via a high-temperature solid-state reaction. Typically, 1 mmol of MgO, Nb_2_O_5_ and Cr_2_O_3_ were weighed and thoroughly ground in an agate mortar for 30 min, followed by the heating treatment at 1450 °C for 6 hours with a heating rate of 5 °C/min. After cooling to room temperature (RT), the resulting pink samples were ground into fine powders for further characterizations. Note that the synthesis procedures of MgNb*_2-x_*Ta*_x_*O_6_:Cr^3+^ crystals were similar except the addition of Ta_2_O_5_ in the first step.

***Preparation of CaZnOS:Yb^3+^ ML crystals:*** According to our previous work, rare-earth ions (i.e., Yb^3+^ or Nd^3+^) doped CaZnOS crystals were synthesized through a high-temperature solid-state reaction under a protection gas flow of N_2_.^[1,2]^ Typically, stoichiometric raw materials and Li_2_CO_3_ flux (10%) were weighed based on the nominal composition of Ca_0.98_RE_0.02_ZnOS and then ground thoroughly in an agate mortar. Subsequently, the mixtures were transferred to alumina crucibles for heating treatment at 1100 °C for 2 hours in a horizontal tube furnace. After cooling to RT, the resulting samples were uniformly ground into fine powders for further characterization.

***Preparation of Lu_3_Al_5_O_12_:Ce^3+^ ML crystals:*** According to the previous report, Lu_3_Al_5_O_12_:Ce^3+^ powder sample was synthesized through a high-temperature solid-state reaction.^[3]^ Typically, stoichiometric raw materials were weighed based on the nominal composition of Lu_2.64_Ce_0.36_Al_5_O_12_ and ground thoroughly in an agate mortar for 30 min. Subsequently, the mixtures were transferred to alumina and then calcined at 1575 °C for 6 hours. After cooling to RT, the resulting samples were uniformly ground into fine powders for further characterization.

***Preparation of Ba_5_(PO_4_)_3_Cl:Eu^2+^ crystals:*** According to the previous report, Ba_5_(PO_4_)_3_Cl:Eu^2+^ powder sample was synthesized through a high-temperature solid-state reaction.^[4]^ Typically, stoichiometric raw materials were weighed based on the nominal composition of Ba_4.9_Eu_0.1_(PO_4_)_3_Cl and ground thoroughly in an agate mortar for 30 min. Subsequently, the mixtures were transferred to alumina and pre-sintered at 400 °C for 1 h in air to decompose the ammonium salts. After grinding into powders, the pre-sintered samples were further sintered in a tube furnace at 1050 °C for 5 h under a gas flow of H_2_/N_2_ (10%/90%). After cooling to RT, the resulting samples were uniformly ground into fine powders for further characterization.

***Preparation of doped Ga_2_O_3_ crystals:*** According to our previous work, impurity-doped Ga_2_O_3_ sample was synthesized through a high-temperature solid-state reaction.^[5]^ Typically, stoichiometric raw materials were weighed based on the nominal composition of Ga_1.98_Cr_0.02_O_3_ and ground thoroughly in an agate mortar for 30 min. Subsequently, the mixtures were transferred to alumina and then calcined at 1100 °C for 5 hours. After cooling to RT, the resulting green samples were uniformly ground into fine powders for further characterization. Note that the synthesis procedures of Ga_1.18_In_0.8_Cr_0.02_O_3_ crystal was similar except the addition of In_2_O_3_ in the first step.

***Preparation of Y_3_Al_5_O_12_:Cr^3+^ crystals:*** According to the previous report, Y_3_Al_5_O_12_:Cr^3+^ powder sample was synthesized through a high-temperature solid-state reaction.^[6]^ Typically, stoichiometric raw materials were weighed based on the nominal composition of Y_3_Al_0.45_Cr_0.05_O_12_ and ground thoroughly in an agate mortar for 30 min. Subsequently, the mixtures were transferred to alumina and then calcined at 1550 °C for 5 hours. After cooling to RT, the resulting samples were uniformly ground into fine powders for further characterization.

***Preparation of Ba_2_ScSbO_6_:Cr^3+^ crystals:*** According to the previous report, Ba_2_ScSbO_6_:Cr^3+^ powder sample was synthesized through a high-temperature solid-state reaction.^[7]^ Typically, stoichiometric raw materials were weighed based on the nominal composition of Ba_2_Sc_0.94_Cr_0.06_SbO_6_ and ground thoroughly in an agate mortar for 30 min. Subsequently, the mixtures were transferred to alumina and then calcined at 1500 °C for 4 hours. After cooling to RT, the resulting samples were uniformly ground into fine powders for further characterization.

***Preparation of composite ML films:*** To evaluate ML performance of fine-powder samples under frictional loads, the ML particles (~ 0.2 g) were placed into a recess (1 × 2 × 0.1 cm) on a nano-adhesive layer affixed to a transparent polyethylene glycol terephthalate (PET) film (4 × 6 cm). This assembly was then encapsulated with another transparent PET film using a thermal laminator to obtain the PET-sealed composite ML film. To assess ML performance of flexible composite elastomer, the curing agent was premixed with polydimethylsiloxane (PDMS) base at a weight ratio of 1:10, followed by the addition of powder sample under vigorous stirring at a weight ratio of 1:2 with PDMS. Then, the mixture was transferred into a rectangle dish (1 × 3 cm) and solidified at 70 ^o^C for 2 hours to get the sample@PDMS film with a thickness of around 50–100 μm. The fabrication procedures for the controlled elastomers composited in polyurethane (PU, Foshan Tongning New Material Co., Ltd) and silicone (SC, Shinbon New Material Co., Ltd) were similar, with the exception that the weight ratios of base resin, curing agent, and powder sample were set as 1:1:1. To examine ML performance of hard pellets under compressive loads, the ML particles (~ 4 g) were thoroughly dispersed with the mixture (~ 10 g) of epoxy resin (ER, Nale New Material Technology Co., Ltd) and curing agent at a weight ratio of 2:3 in a plastic mold (diameter ~ 30 mm, thickness ~ 10 mm), followed by the solidification at 60 ^o^C for 4 hours. After cooling to RT, the pellet was demolded to get the sample@ER hard pellet.

***Preparation of ML ceramics:*** Polyvinyl alcohol (PVA) was used as the binder to prepare molded powder sample with high surface flatness. First, fine powders (~ 0.1 g) were thoroughly mixed with PVA at a weight ratio of 100: 1 to form homogeneous slurry, which was then pressed into a dense disc using a cylindrical mold under a hydraulic press. Then, the disc was calcined at 1450 ^o^C for 6 hours in ambient air. Finally, the disc surface was polished with a polish-grinding machine (MPD-1S, Microcre Optics-Mech Tech Co., Ltd) to achieve a roughness of 10–200 nm.

***General characterization:*** Powder X-ray diffraction (PXRD) measurement was conducted by a Bruker D8 advance powder diffractometer using Cu-Kα (*λ* = 1.54056 Å) irradiation. The refinement results were acquired using the software of General Structure Analysis System (GASA-II). The microstructural information, including scanning electron microscopy (SEM), energy dispersive spectrometer (EDS), high-resolution transmission electron microscopy (HR-TEM), and selected area electron diffraction (SAED), was characterized by a Novanano-450 field SEM and a JEOL JEM-F200 TEM. X-ray photoelectron spectroscopy (XPS) was measured by a Thermo Fisher ESCALAB 250Xi, where binding energies were all calibrated using the C 1s peak at 284.6 eV. The converse piezoelectric coefficient (*d*_33_^*^) was assessed based on piezoresponse force microscopy (PFM) measurements using an atomic force microscope (Oxford MFP-3D Origin+) in contact mode. Note that the ML particles were ultrasonically dispersed in ethanol and drop-cast onto a silicon wafer prior to the measurement. Raman and diffuse reflection (DR) spectra were measured using a HR Evolution confocal Raman microscopy and a Hitachi 4100 UV-Vis-NIR spectroscopy, respectively. The photoluminescence property, including PL excitation (PLE), emission spectra, PL lifetimes, and persistent luminescence (PersL) decay curves were recorded using a Horiba FL3 fluorescence spectrometer equipped with a 450 W xenon lamp as the excitation source. PL quantum yield (PLQY) was measured by a Hamamatsu C9920-02 PLQY spectrometer. Cathodoluminescence (CL) spectra were measured using the FEI Quanta 200 ESEM and Goldenscope Rainbow. Mechanoluminescence (ML) spectra were captured by an Ocean Optics QEPRO fiber optic spectrometers. The optical images were taken by an iPhone 13 and an ORPHA ONV3+ night-vision monocular. The triboelectric potential was measured using an electrostatic probe (SK-1000, KEYENCE Co., Ltd.) after cyclic rubbing between ML ceramics and organic film.

***Quantitative determination of ML performance:*** The ML performance under frictional loads was quantitatively assessed by a homemade device.^[8]^ A customized mechanical control unit, comprising a digital push-pull gauge, a linear translation stage, and a linear/rotating motor, was employed to apply quantitative frictional stimulation. By screwing the micrometer on the translation stage, the relative position between the force gauge and sample holder can be finely adjusted to control the magnitude of the force. The displacement of the force gauge along the linear/circular track resulted in the sliding of its metal indenter over the film to generate ML. Note that the surface triboelectric potential can be simultaneously measured using a nearby electrostatic probe. In addition, the compressive and stretching loads were achieved using a universal testing machine (CTM2050, Xieqiang Instrument Manufacturing Co., Ltd.). With these systems established, ML spectra were captured by a nearby fiber-coupled charge-coupled device (CCD) spectrometer. Meanwhile, ML intensity was recorded over time by extracting the grayscale of NIR images taken by a night-vision monocular. The ultrasonically induced ML was measured in an ultrasonic cabinet at various powers. Notably, all the error bars of ML intensity represent the standard deviations from three sets of repeated measurements. In addition, the ML decay curves were measured by a homemade system consisting of a photomultiplier tube (PMT, R928, Hamamatsu), a fast amplifier (C7319, Hamamatsu), and an oscilloscope with bandwidth of 300 MHz (RTB2004, R&S). Note that the ML particles were mixed homogeneously with ER with a mass ratio of 2:1, followed by deposition on a transparent sapphire wafer (diameter ~ 40 mm, thickness ~ 1.28 mm) using a blade-coating method. After being cured at 70 ^o^C for 4 hours, a tough ML film (thickness ~ 0.1 mm) was obtained on the sapphire wafer for ML decay measurement.

***Computation details:*** The theoretical calculations were performed by the Vienna Ab Initio Simulation Package (VASP). The formation energy was calculated by density functional theory (DFT) in the form of generalized gradient approximation (GGA) Perdew-Burke-Ernzerhof (PBE) function, and the HSE06 generalized functional was employed for the band structure, and partial density of states (PDOS).^[9,10]^ A Monkhorst-Pack 4 × 4 × 4 k mesh was used as Brillouin zone. The kinetic energy cutoff and self-consistent field (SCF) were set as 500 eV and 10^-5^ eV/atom, respectively.


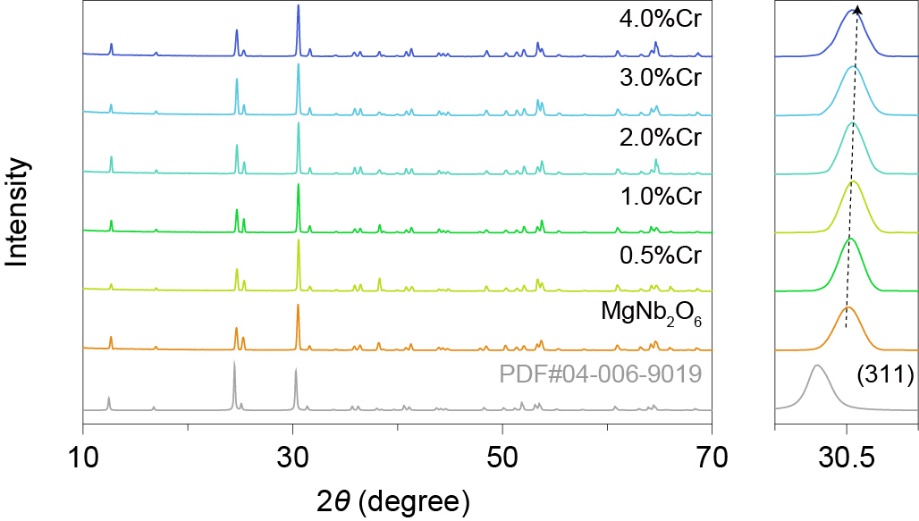


**Figure S1.** XRD patterns of MgNb_2_O_6_:*x*Cr^3+^ (*x* = 0, 0.5%, 1%, 2%, 3%, 4%) crystals, along with the magnified diffraction peaks of (311). Notably, the continuous red-shift of diffraction peaks indicated a steady shrinkage of unit cell as increasing Cr^3+^ doping concentration.


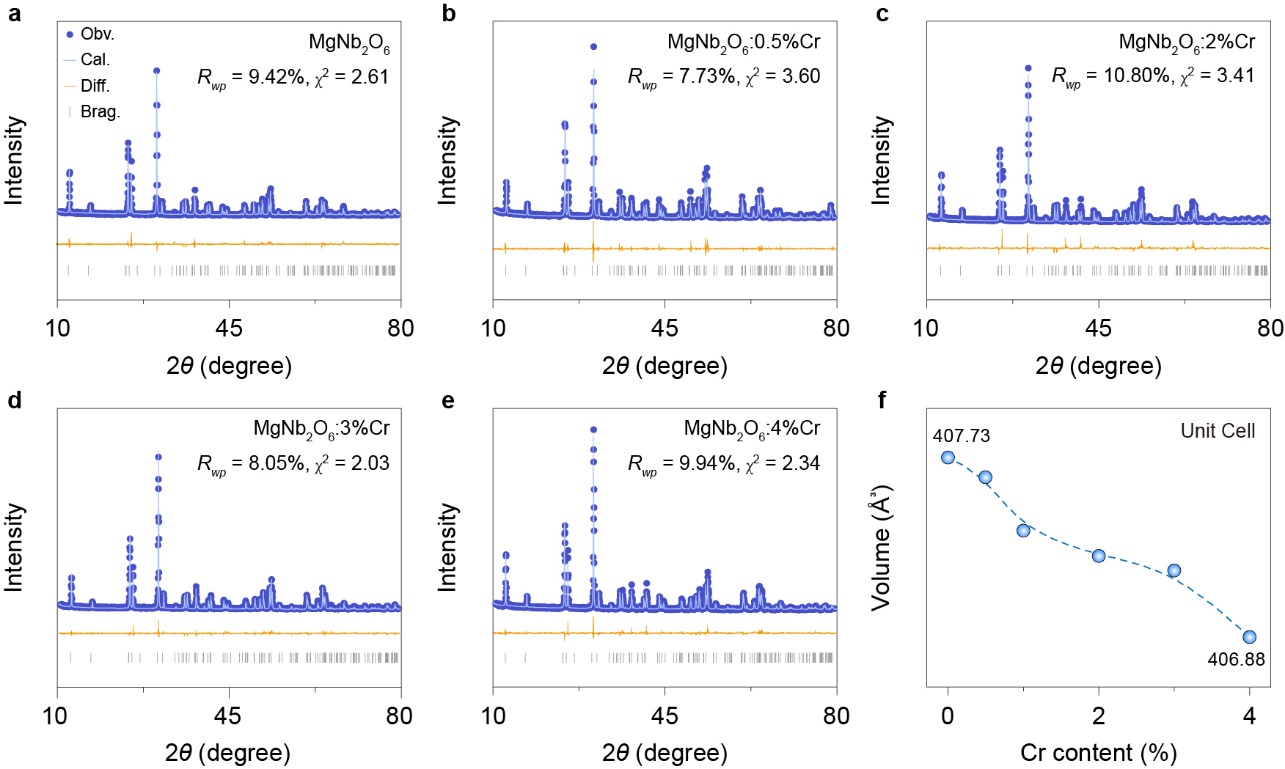


**Figure S2. a-e)** Rietveld refinements of XRD patterns for MgNb_2_O_6_:Cr^3+^ crystals, and **f)** the calculated cell volumes as a function of Cr^3+^ doping concentration. Notably, the cell volume was gradually decreased as increasing Cr^3+^ content, revealing the successful replacement of Mg^2+^ (*r* = 0.72 Å) by smaller Cr^3+^ (*r* = 0.615 Å). The detailed crystallographic structural parameters are summarized in **Table S2**.


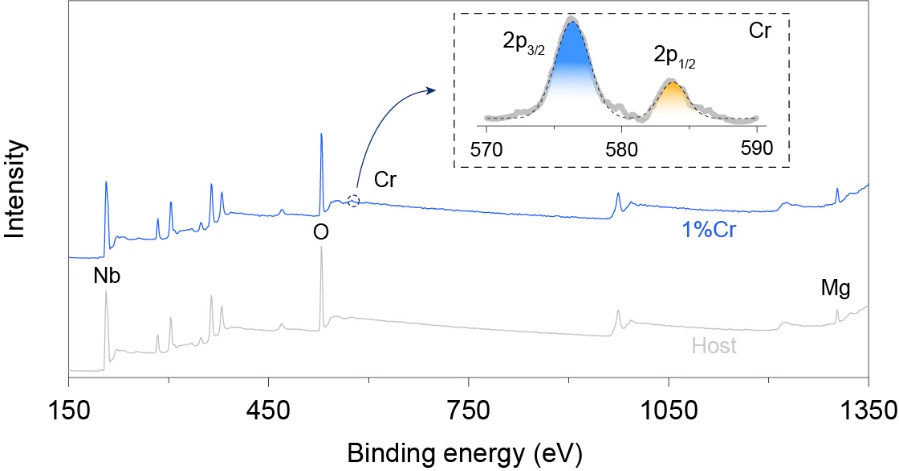


**Figure S3.** Typical XPS survey spectra of the bare and Cr^3+^-doped MgNb_2_O_6_ crystals, and the inset shows high-resolution Cr-2p XPS survey. It can be found that binding energy information of Mg, Nb, and O elements was detected in these crystals. After Cr ion doping, two additional peaks at 576.3 and 585.9 appeared corresponding to Cr^3+^: 2p_3/2_ and 2p_1/2_ doublets, confirming the presence of trivalent chromium without a secondary valence state.


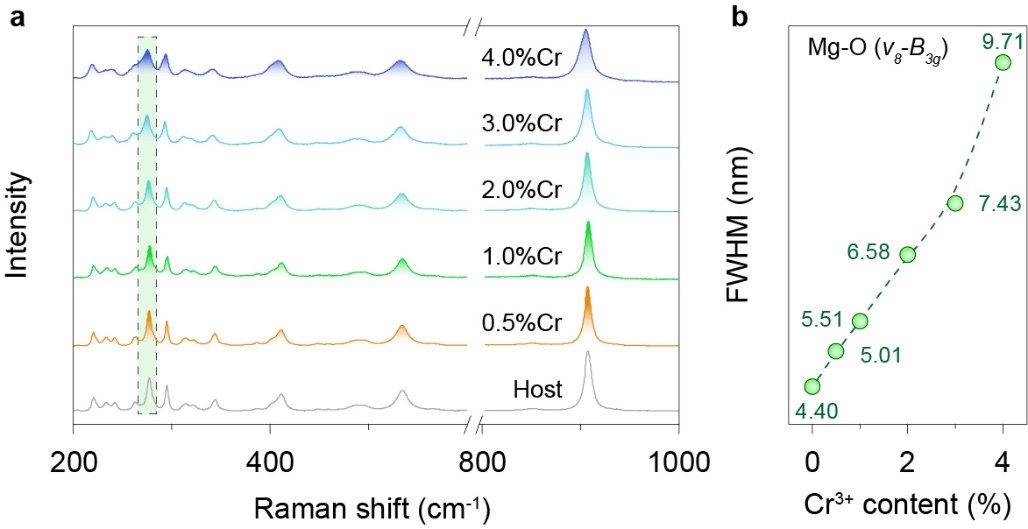


**Figure S4. a)** Raman spectra and **b)** the full width at half maximum (FWHM) values at 277.3 cm^-1^ of MgNb_2_O_6_:*x*Cr^3+^ (*x* = 0, 0.5%, 1%, 2%, 3%, 4%) crystals. Notably, the linewidth of the Raman band (Mg-O, *υ*_8_-*B*_3g_) broadened significantly upon Cr^3+^ doping due to the local symmetric distortion, further confirming the preferential substitution of Cr^3+^ in Mg^2+^ sites.


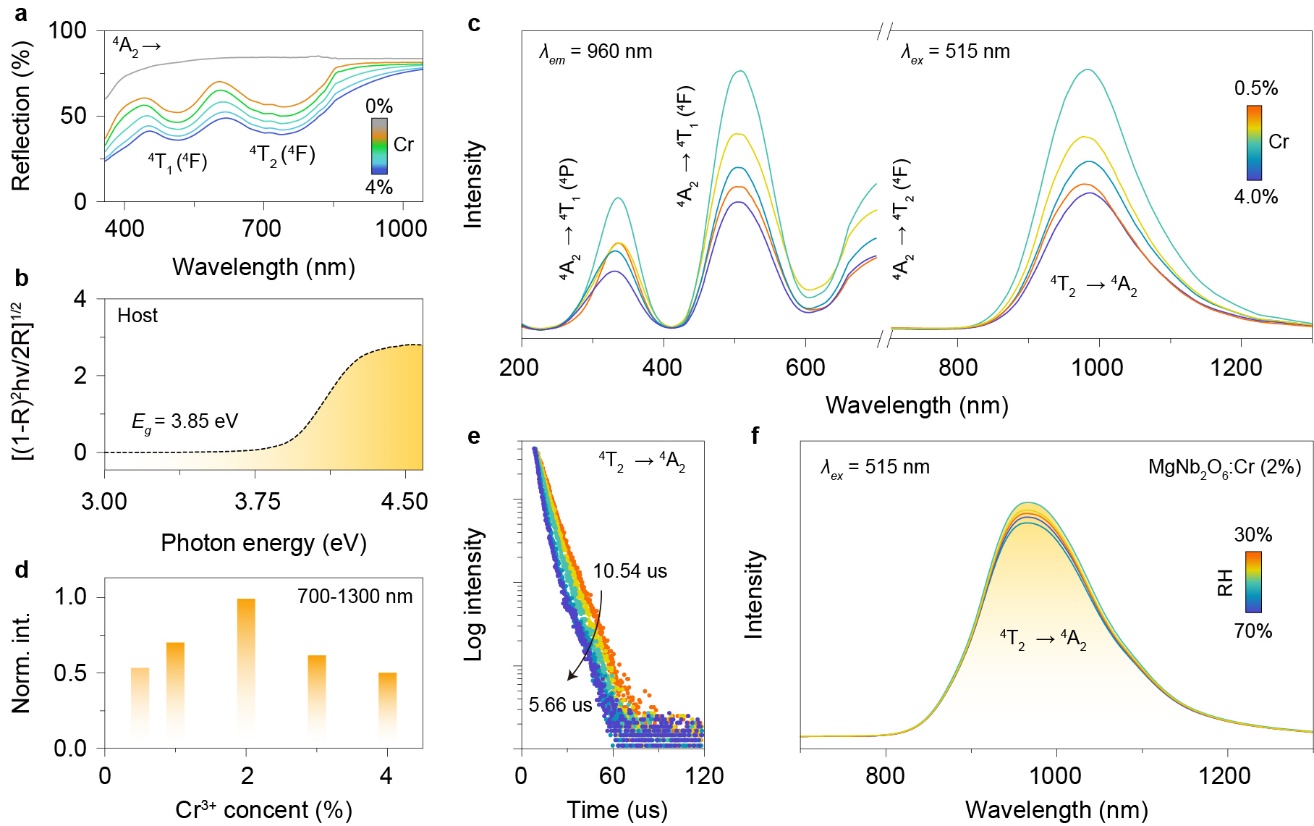


**Figure S5. a)** DR spectra of MgNb_2_O_6_:*x*Cr^3^⁺ (*x* = 0–4%) in the range of 350–1050 nm. Notably, only the characteristic Cr^3+^ peaks were detectable, with no peaks from Cr^4+^ ion. Based on the peak energy, the crystal field parameters (*Dq*/*B*) can be calculated by using formulas proposed by Henry, Tanabe, and Sugano:^[11]^

$E({}^{4}T_{2})=10\text{Dq}$ (S1)

$\text{E(}{}^{4}\text{T}_{1}\text{)}=15Dq+7.5B-0.5\sqrt{(10Dq)^{2}-18Dq\times B+255B^{2}}$ (S2)

where *Dq* and *B* represent the crystal field splitting energy and Racha parameter, respectively. The *Dq*/*B* value was calculated to be around 2.1 and slightly affected by the Cr^3+^ content, indicating a weak crystal field in octahedral Cr^3+^. **b)** The calculated Kubelka-Munk absorption spectrum of the host material, with the calculated optical bandgap of around 3.85 eV. **c)** PLE (*λ*_em_ = 960 nm) and PL spectra (*λ*_ex_ = 515 nm) of MgNb_2_O_6_:*x*Cr^3^⁺ (*x* = 0–4%) crystals. **d,** Integral emission intensity (700–1300 nm) as a function of dopant concentration. **e)** Decay curves of ^4^T_2_ → ^4^A_2_ transition at 960 nm. With the increase of Cr^3+^ content from 0.5% to 4%, the lifetime fitted by a single-exponential function gradually decreased from 10.54 to 5.66 μs due to the promoted non-radiative process. **f,** PL spectra of MgNb_2_O_6_:Cr^3^⁺ (2%) in ambient air with different relative humidity (~ 30–70%). Note that a humidifier was applied to approximately control the air humidity around the sample during the PL measurements.


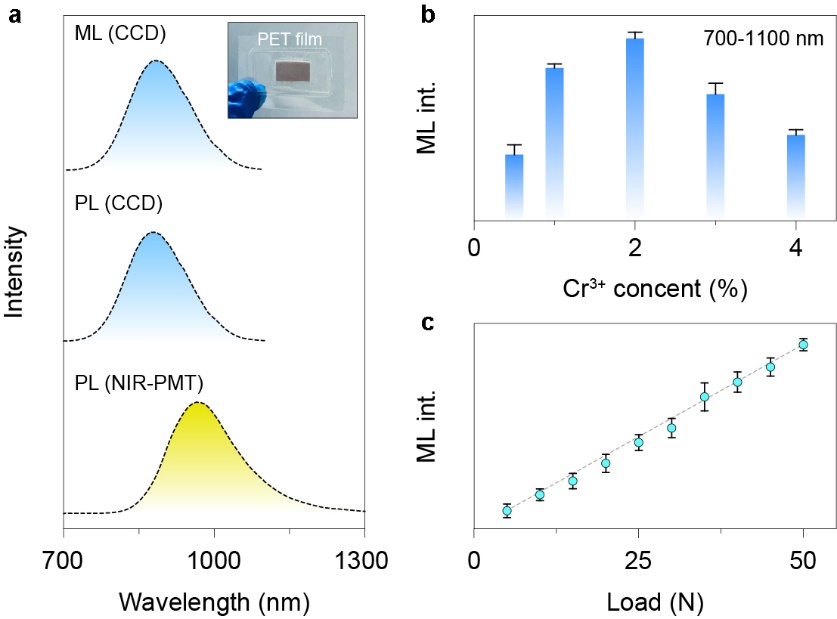


**Figure S6. a)** The spectral comparison between NIR PMT and CCD detectors, and inset shows a photograph of PET-sealed ML sample. Compared with the NIR-PMT detector (600–1700 nm), the spectral response of the CCD detector (200–1100 nm) drops greatly over 750 nm, leading to degradation in spectral performance. As a result, the central wavelength (~ 875 nm) recorded by a CCD detector was shorter than that measured by NIR-PMT (~ 960 nm). It is worth noting that the actual ML spectrum should align with PL spectrum obtained using the NIR-PMT. **b)** Integral ML intensity (700–1100 nm) of MgNb_2_O_6_:*x*Cr^3^⁺ (*x* = 0–4%) crystals under mechanical excitation at 50 N. The optimal Cr^3+^ concentration was determined to be 2% with the strongest ML intensity, consistent with PL results. **c)** Integral ML intensity of MgNb_2_O_6_:Cr^3^⁺ (2%) as a function of applied force (5–50 N). With the increase of frictional loads, the PET-sealed powder sample showed a nearly linear ML enhancement, highlighting the promise for stress sensing applications.


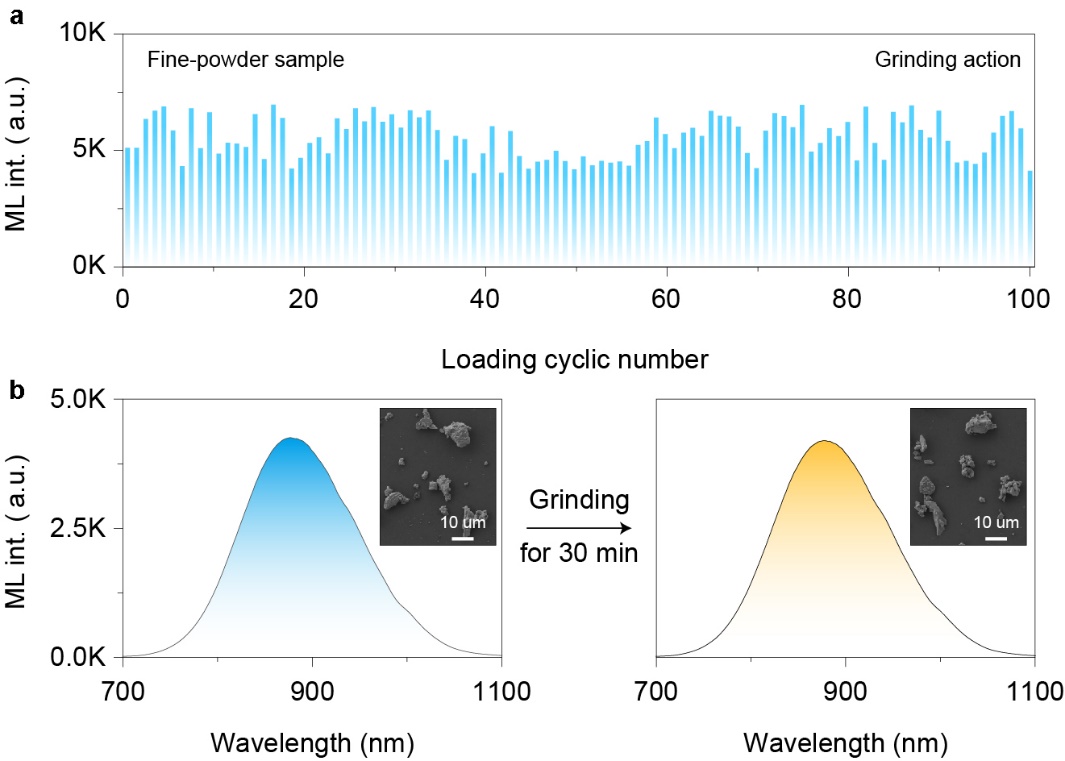


**Figure S7. a)** The cyclic stability of MgNb_2_O_6_:Cr^3^⁺ (2%) under continuous grinding. An optical fiber was inserted and fixed into a glass tube to apply a sliding force on the fine-powder sample. The force was carefully controlled to maintain consistency, though minor variation was inevitable. It can be found that the ML intensity hardly attenuated during continuous grinding action. **b)** ML spectra and SEM images of MgNb_2_O_6_:Cr^3^⁺ fine-powder sample before and after grinding for 30 minutes. Note that the as-prepared sample was first ground into fine powders prior to measurements. The grinding action hardly affected the morphology, grain size, and ML intensity of the original microparticles, thereby ruling out the main contribution of the fracture-induced ML mechanism. These results confirm the self-recoverable nature of ML in MgNb_2_O_6_:Cr^3^⁺ crystal.


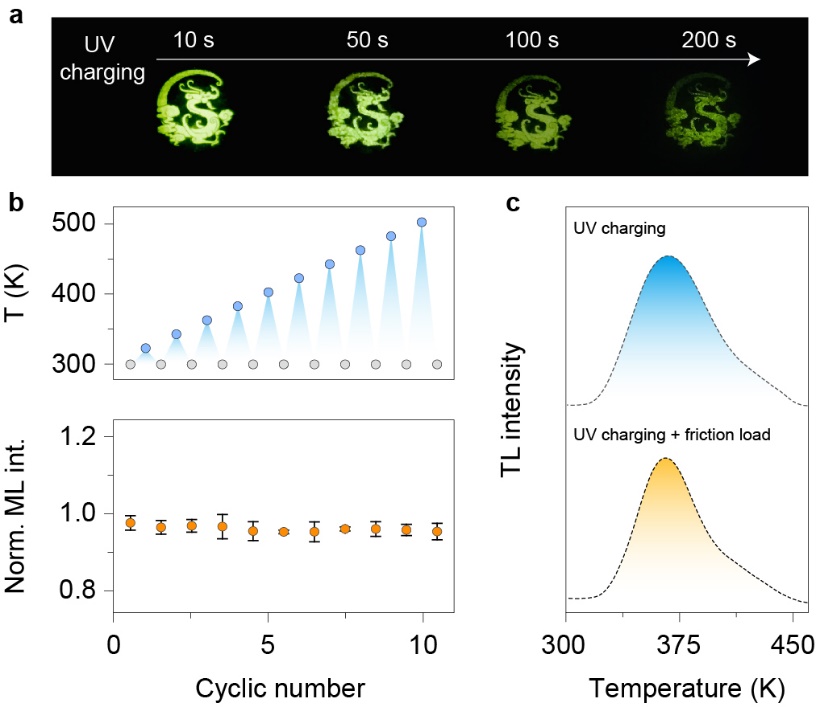


**Figure S8. a)** SWIR PersL images of MgNb_2_O_6_:Cr^3^⁺ (2%) powder after 254 nm charging for 5 minutes recorded by a night-vision monocular. **b)** The cyclic stability of ML intensity at RT after heating treatments at different temperatures (300–500 K). Notably, the ML intensity remained nearly unaltered after depleting the energy stored in trap states via thermal bleaching pre-treatment at various temperatures. **c)** TL spectra of MgNb_2_O_6_:Cr^3^⁺ (2%) recorded after 254 nm charging with or without friction action. The sample was first charged by a 254 nm light for 5 minutes, followed by either placing or grinding for 5 minutes before homemade TL measurements. Accordingly, TL spectra remained essentially unchanged before and after force stimulation, suggesting that ML photons are unlikely to originate from the PersL-involved trap states. These results suggest that the widely reported PersL-controlled ML model can be ruled out in the present material system.


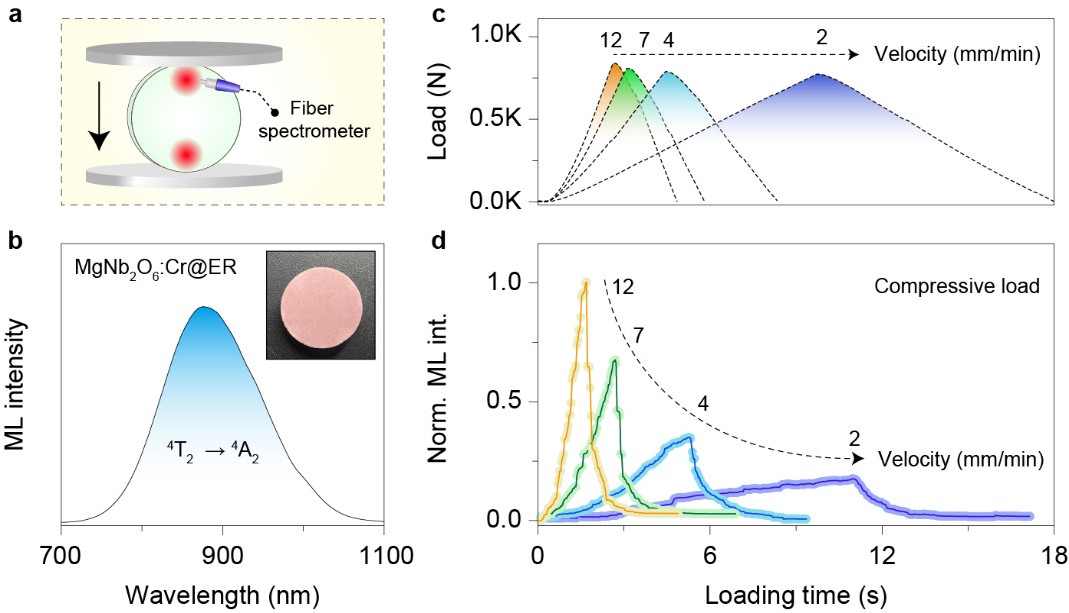


**Figure S9. a)** Schematic of the ML measurement setup under compressive loads. A universal testing machine was used for applying dynamic compressive loads on hard ML pellets, while the optical signal was captured by a nearby fiber-coupled spectrometer and a night-vision monocular. **b)** ML spectrum of MgNb_2_O_6_:Cr^3^⁺ (2%) embedded in ER polymers under compressive load at 800 N, and the inset shows the photograph of the MgNb_2_O_6_:Cr^3^⁺@ER pellet. **c)** The evolution of applied compressive load (0–800 N) as a function of the loading time at varied velocities (2–12 mm/min) during a compression-release cycle. Note that the compressive and release velocities were set to the same. **d)** The variation of instantaneous ML intensity versus the loading time. Notably, the ML intensity was positively correlated with the applied compressive velocity, but rapidly decayed during release irrespective of the release velocity. These results suggest that the ML only responds to dynamic compression, rather than to release or static loads.


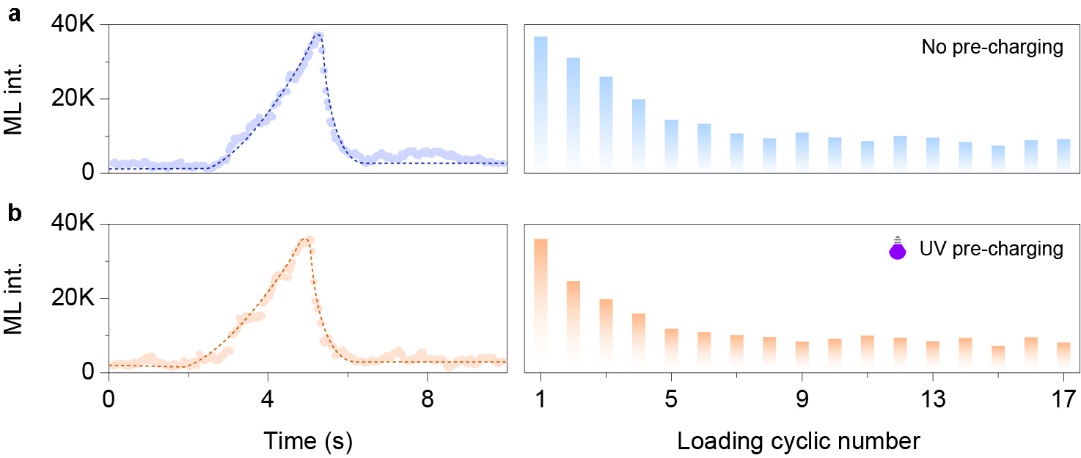


**Figure S10.** Time-resolved ML intensity curve during a compression-release cycle and cyclic stability under repeated compressive load (800 N, 7 mm/min) of MgNb_2_O_6_:Cr^3^⁺@ER pellet **(a)** with and **(b)** without 254 nm pre-charging for 5 minutes. There was no noticeable difference in ML intensity and repeatability before and after UV charging, further ruling out the contribution of trap states to the ML process.


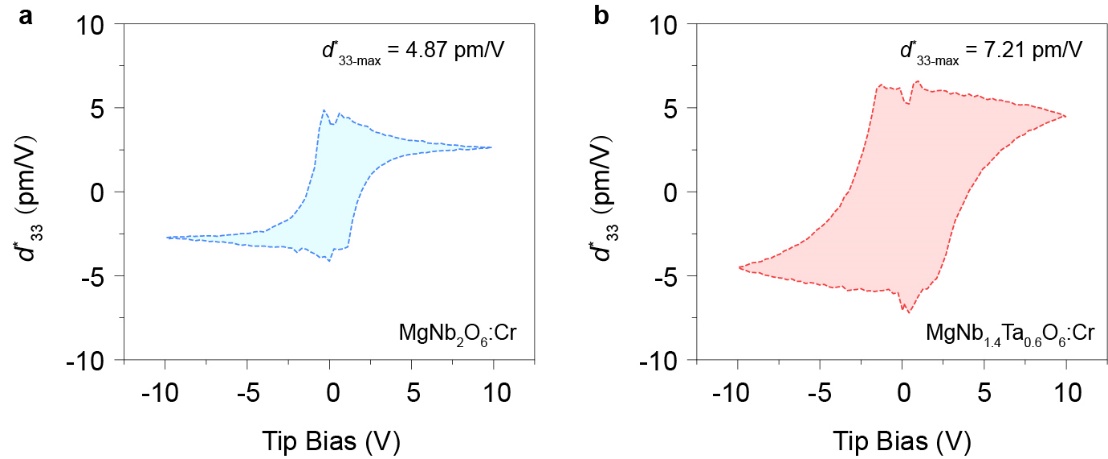


**Figure S11.** The converse piezoelectric coefficient of **a)** MgNb_2_O_6_:Cr^3^⁺ and **b)** MgNb_1.4_Ta_0.6_O_6_:Cr^3+^ powders derived from PFM measurements under a tip bias in the range of ± 10 V. Based on the converse piezoelectric effect, the relationship between displacement (*D*) and applied voltage (*V*) can be expressed as:^[12]^

$\text{D}\text{ }\text{=}\text{ }\text{d}_{\text{3}\text{3}}^{\text{*}}\text{ }\text{×}\text{ }\text{V}$ (S3)

where *d*_33_^*^ is the converse piezoelectric coefficient. Taking the shift of the intersection of the butterfly curve from the origin point into consideration, the equation S3 can be modified as:^[12]^

$\text{d}_{\text{3}\text{3}}^{\text{*}}\text{ }\text{=}\text{ }\frac{\text{D - }\text{D}_{0}}{\text{V - }\text{V}_{0}}$ (S4)

where *D*_0_ and *V*_0_ are the displacement and voltage of the intersection, respectively. In this case, a larger amplitude represents a higher *d*_33_^*^ value. Notably, the maximum value of *d*_33_^*^ was substantially enhanced from 4.87 to 7.21 pm/V upon Ta^5+^ doping, in consistency with the ML enhancement.


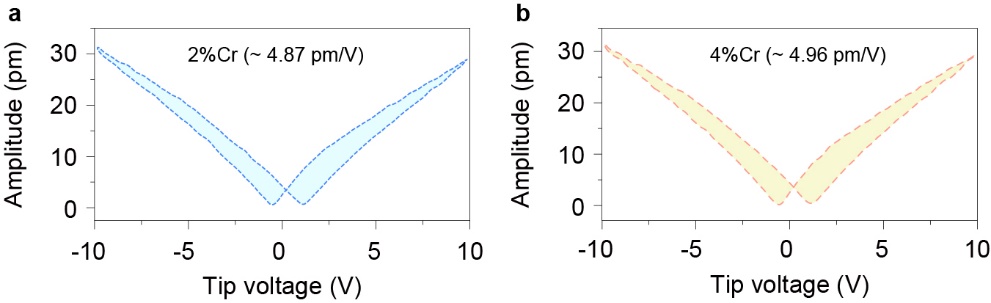


**Figure S12**. PFM amplitude of MgNb_2_O_6_:Cr^3+^ (2%, 4%) under a tip bias in the range of ± 10 V. Notably, the observed amplitude butterfly loop indicated the presence of piezoelectric effect in these two samples. The increase of Cr^3+^ concentration hardly affected the piezoelectric response, indicating that the local piezoelectric behavior remained largely unchanged. Therefore, the decline in ML intensity at heavy Cr^3+^ concentration (> 2%, **Figure S6b**) originates primarily from concentration quenching, rather than from changes in piezoelectric properties.


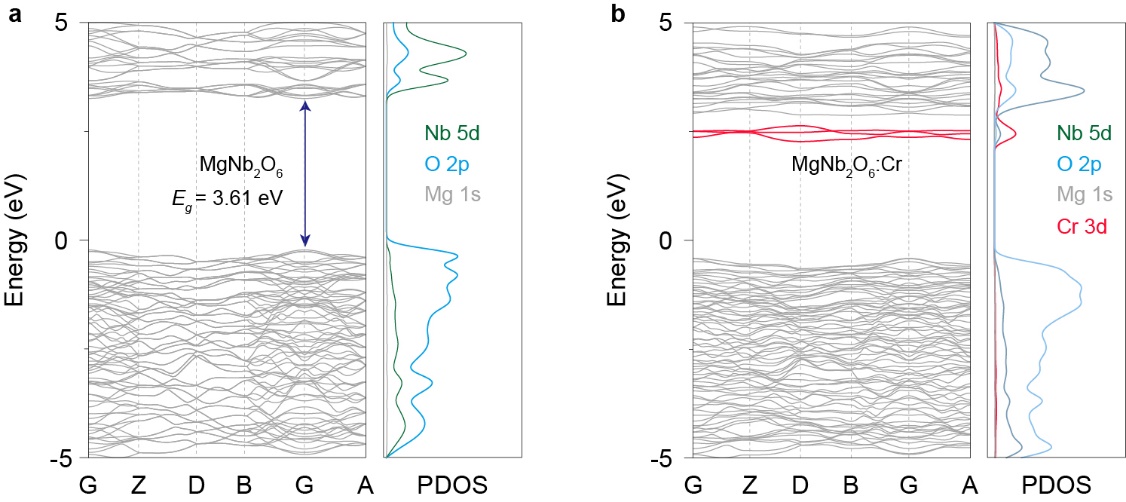


**Figure S13.** The calculated electronic band structures and partial density of states (PDOS) of **a)** the bare and **b)** Cr^3+^-doped MgNb_2_O_6_ crystals. Specifically, the band structure of the bare crystal features a direct bandgap of 3.61 eV at the G point, in close agreement with the experimental value (~ 3.85 eV). As revealed by the PDOS, the Nb 5d orbitals primarily constitute the bottom of the conduction band (CB), while the O 2p orbitals dominate the top of the valence band (VB). After the introduction of Cr^3+^ ion, the original local electronic structures are modified by forming inter-electronic 3d levels near the CB bottom. Therefore, the breakdown of the local inversion symmetry caused by uneven electronic distribution possibly enables centrosymmetric crystals to generate localized piezoelectric fields in response to external stress.


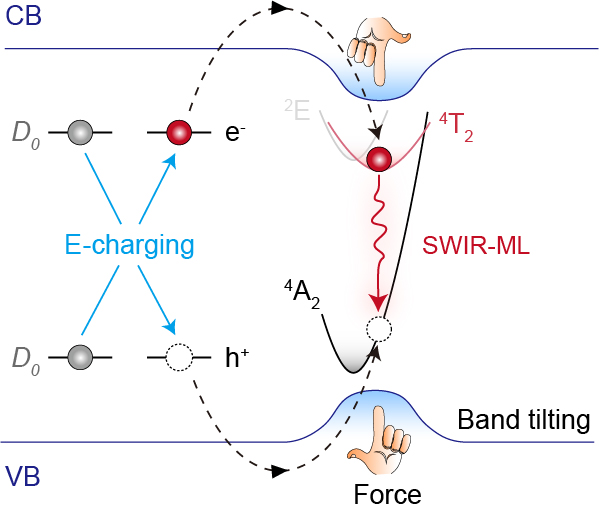


**Figure S14.** Schematic illustration of the proposed ML mechanism in MgNb_2_O_6_:Cr^3^⁺ crystal. *D_0_*: neutral defect centers, e^-^: electron, h^+^: hole, *E*-charging: local piezoelectric field-induced separation of charge carriers. The possible ML process at the microscopic level can be proposed as follows.^[5]^ Under mechanical stress, the doping-induced perturbation of inversion symmetry gives rise to a localized piezoelectric field, accompanied by energy band tilting. Driven by the inner piezo-potential, charge carriers are released and separated from neutral defect centers, followed by radiative recombination through the Cr^3+^ levels to produce broadband SWIR-ML.

**
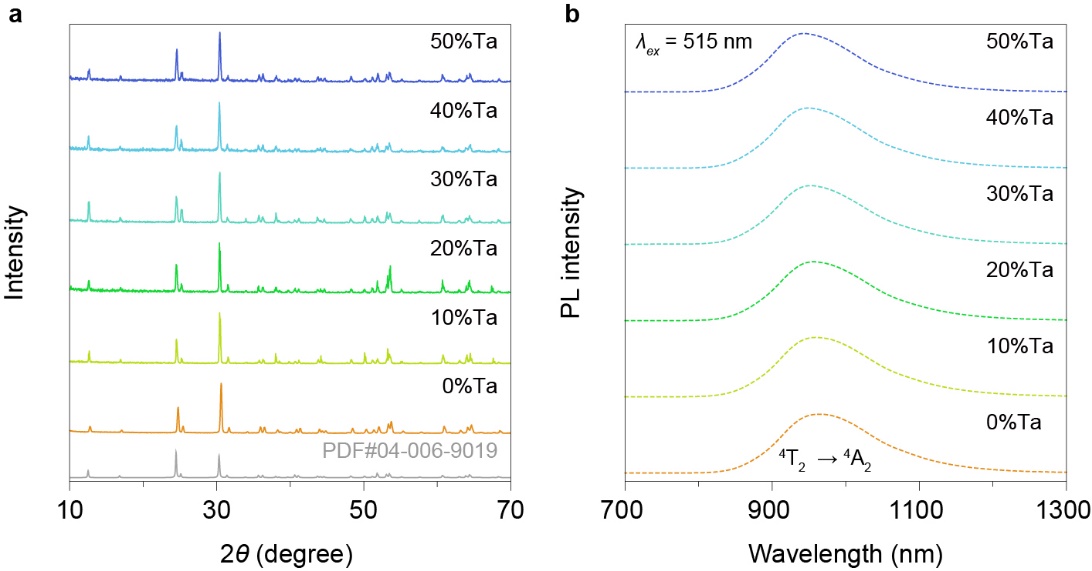
**

**Figure S15. a)** XRD patterns and **b)** PL spectra (*λ*_ex_ = 515 nm) of MgNb_2_O_6_:Cr^3^⁺/*x*Ta^5+^ (2%/*x* = 0, 10%, 20%, 30%, 40%, 50%) crystals. Notably, homogeneous single-phase Mg(Nb/Ta)_2_O_6_ solid solutions were formed without noticeable spectral shifts as elevating Ta^5+^ doping levels. These results can be ascribed to the structural consistency between MgNb_2_O_6_ and MgTa_2_O_6_, as they share the orthorhombic structure with a common space group of *Pbcn*.

**
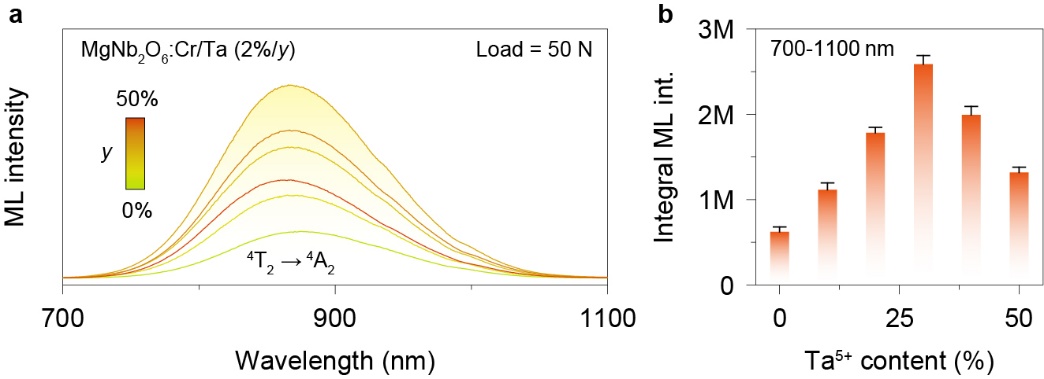
**

**Figure S16. a)** ML spectra and **b)** integral intensity (700–1100 nm) of MgNbO_6_:Cr^3^⁺/Ta^5+^ (2%/*y* = 0–50%) crystals under mechanical excitation at 50 N. Notably, the cation substitution substantially enhanced ML intensity, registering a maximum enhancement factor of 4.2 folds at an optimal Ta^5+^ content of 30%.

**
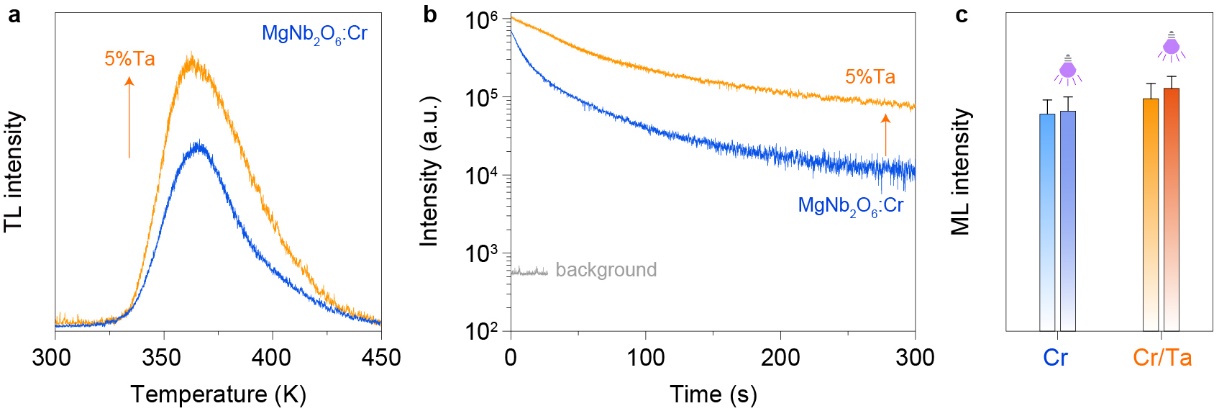
**

**Figure S17. a)** TL spectra and **b)** PersL decay curves of MgNb_2_O_6_:Cr^3+^ (2%) and MgNb_2_O_6_:Cr^3+^/Ta^5+^ (2%/5%) recorded after 254 nm charging for 5 minutes. Notably, the introduction of Ta^5+^ led to an obvious increase in PersL performance, confirming the effective regulation of crystal defects. **c)** Comparison of ML integral intensity with and without UV light pre-charging. Upon Ta^5+^ doping, the ML performance featured only a marginal enhancement, both with and without UV pre-charging, indicating the independent nature of ML behavior on PersL-related defects in MgNb_2_O_6_.

**
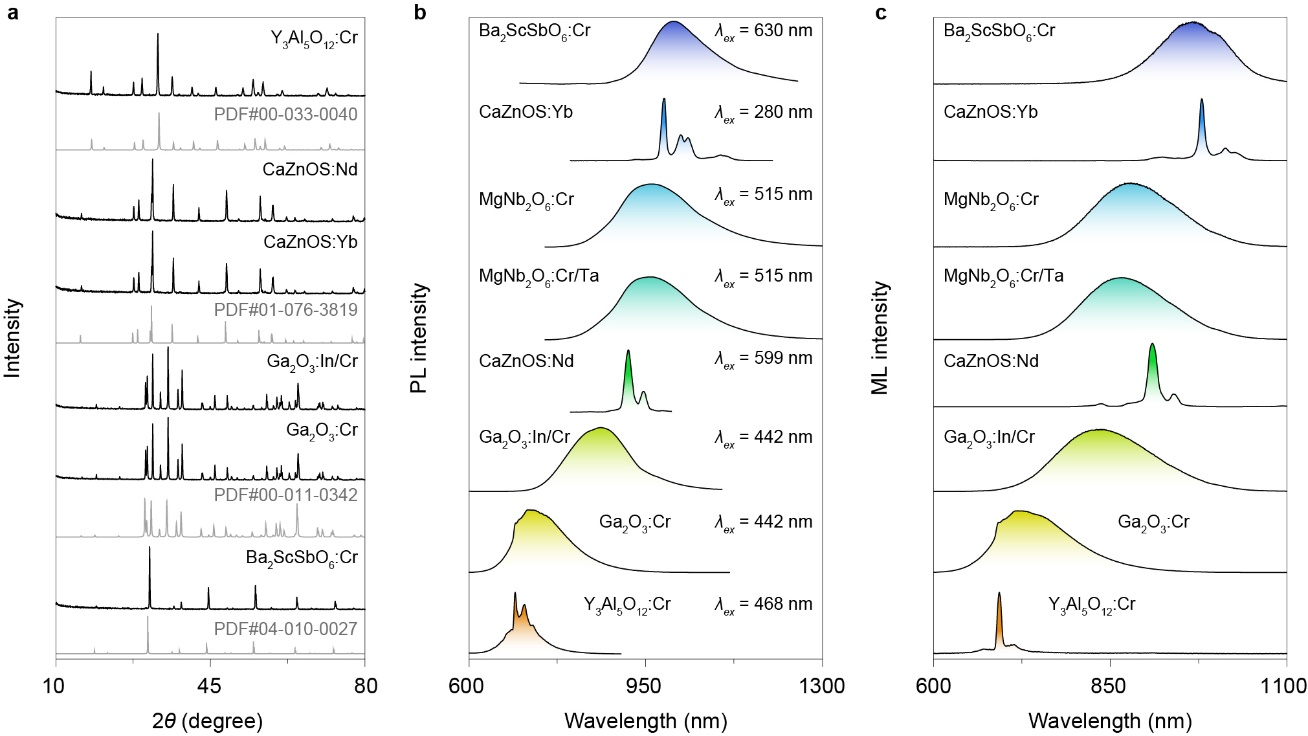
**

**Figure S18. a)** XRD patterns of reported NIR-ML materials, including Ba_2_ScSbO_6_:Cr^3+^ (6%), Ga_2_O_3_:Cr^3+^ (1%), Ga_2_O_3_:Cr^3+^/In^3+^ (1%/40%), CaZnOS:Yb^3+^ (2%), CaZnOS:Nd^3+^ (1%), and Y_3_Al_5_O_12_:Cr^3+^ (1%) prepared by a high-temperature solid-state method (**see** experimental sections). **b)** PL and **c)** ML spectra of these materials, along with MgNb_2_O_6_:Cr^3^⁺ (2%) and MgNb_2_O_6_:Cr^3^⁺/Ta^5+^ (2/30%) crystals. Note that the ML spectra of PET-sealed samples were measured under the identical experimental conditions (50 N). Remarkably, MgNb_1.4_Ta_0.6_O_6_:Cr^3+^ delivered strongest ML among these NIR-ML phosphors.

**
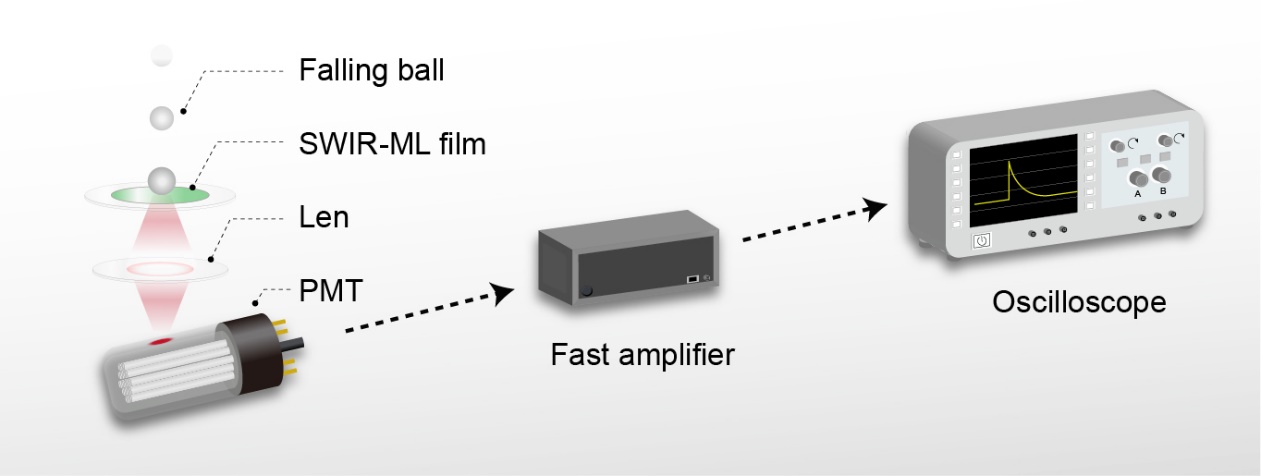
**

**Figure S19.** Schematic illustration of the homemade apparatus for single-shot ML decay measurement.^[13]^ In our design, a free-falling iron ball is applied to generate nondestructive impact excitation on the rigid ML film deposited on a sapphire wafer, where the optical signals are collected by a PMT and transmitted to a digital oscilloscope through a fast amplifier with a scanning rate of 3.2 ns per frame.

**
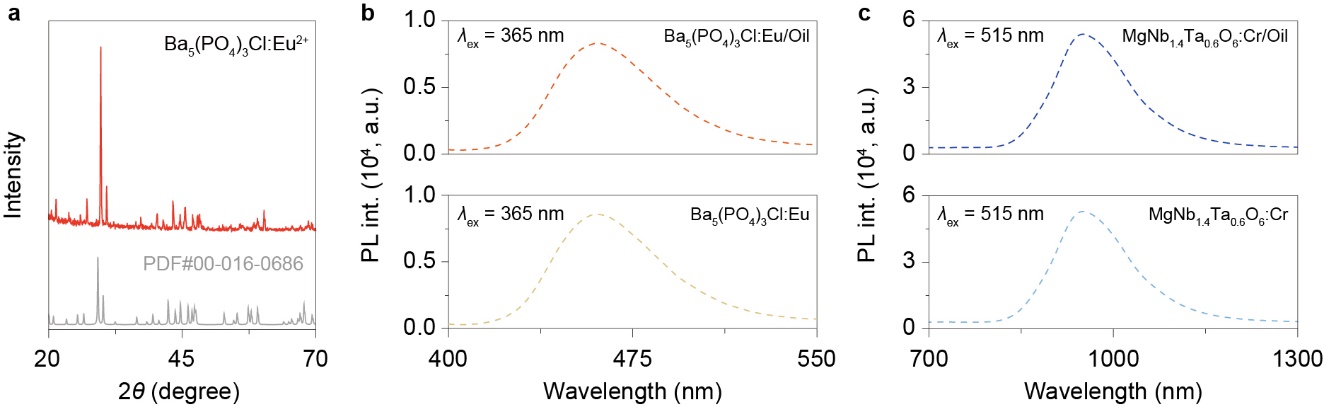
**

**Figure S20.** **a)** XRD patterns of triboelectric ML phosphor Ba_5_(PO_4_)_3_Cl:Eu (10%). **b-c)** PL spectra of Ba_5_(PO_4_)_3_Cl:Eu and MgNb_1.4_Ta_0.6_O_6_:Cr^3+^ fine powders before and after mixing with the lubricant oil. Unlike the ML process, the addition of lubricant oil exerted negligible influences on the PL performances for both crystals. These results provide strong evidence that the triboelectric effect plays a crucial role in driving the ML process in flexible elastomers.

**
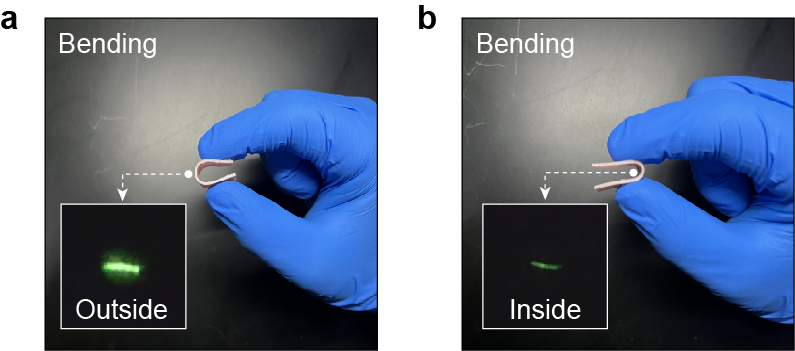
**

**Figure S21.** ML images of MgNb_1.4_Ta_0.6_O_6_:Cr^3+^@PDMS film under bending deformation recorded from **a)** the outer and **b)** inner surface. Notably, the ML can be detected from both the inner and outer sides of the film, corresponding to the compressed and stretched strains, confirming the contribution of stress-induced piezoelectric effect to the ML process.

**
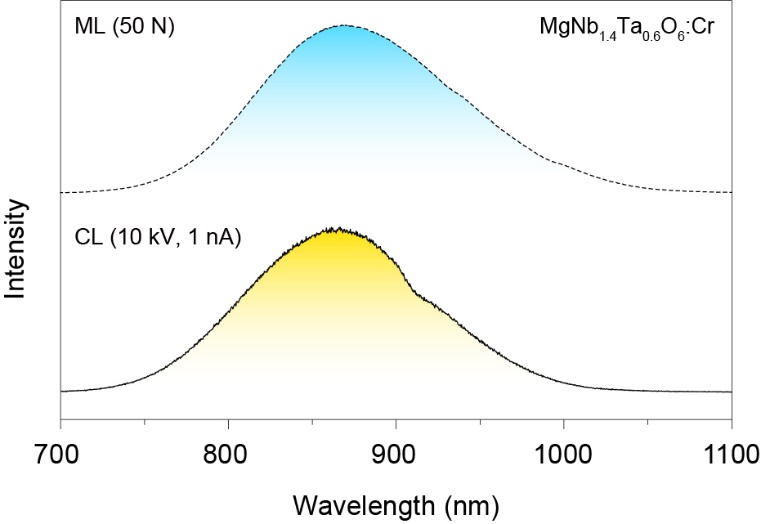
**

**Figure S22.** ML (top column) and CL (bottom column) spectra of MgNb_1.4_Ta_0.6_O_6_:Cr^3+^ (2%) crystal. Notably, the CL spectrum under high-field electron bombardment was basically consistent with the ML one. We thus assume that the impact excitation model might be one of the reasonable theories to explain the ML process in flexible elastomers.

**
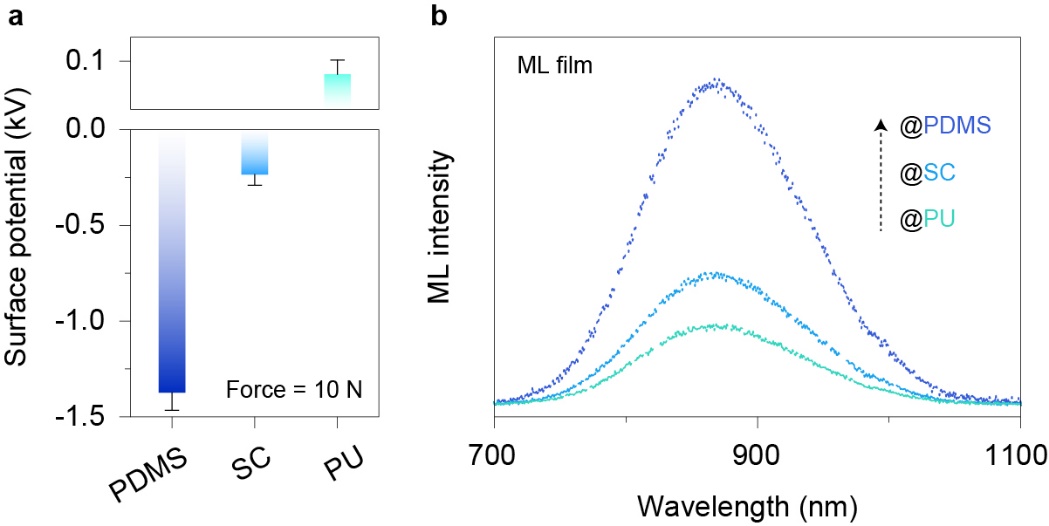
**

**Figure S23.** **a)** Triboelectric potential of the ML ceramics and different organic films (PDMS, SC, and PU) following 1 min of reciprocating friction under a load of 10 N. **b)** ML spectra of MgNb_1.4_Ta_0.6_O_6_:Cr^3+^ (2%) embedded in different organic phases under identical conditions. It is noteworthy that the ML intensity of the composite films exhibits a positive correlation with the surface negative potential of these organic phases. Additionally, the observed ML in phosphor@PU film mainly arises from the piezoelectric effect due to the positive potential of PU.

**
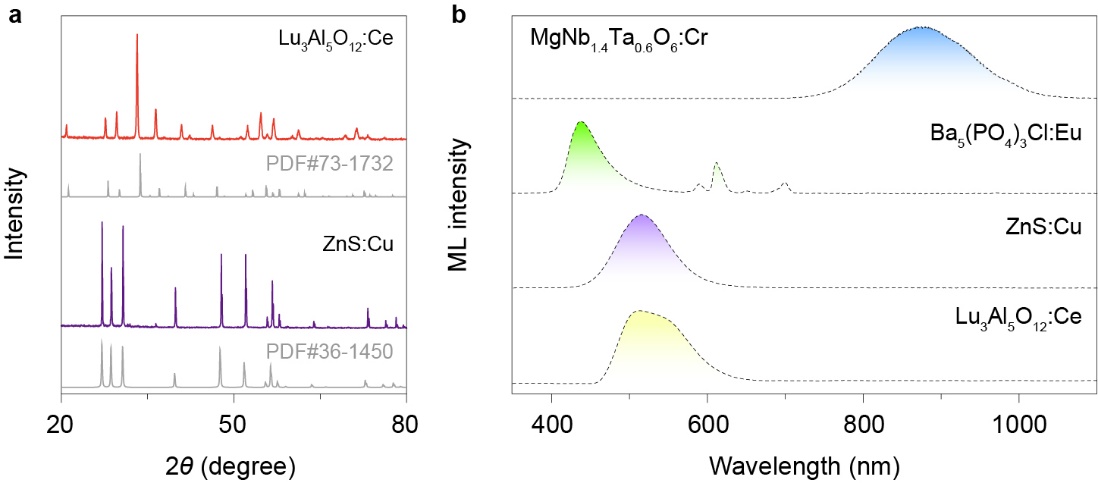
**

**Figure S24.** **a)** XRD patterns of triboelectric ML phosphors ZnS:Cu and Lu_3_Al_5_O_12_:Ce^3+^ prepared by high-temperature solid-state method. **b)** ML spectra of Lu_3_Al_5_O_12_:Ce^3+^, ZnS:Cu, Ba_5_(PO_4_)_3_Cl:Eu^2+^, and MgNb_1.4_Ta_0.6_O_6_:Cr^3+^ films under mechanical excitation.

**
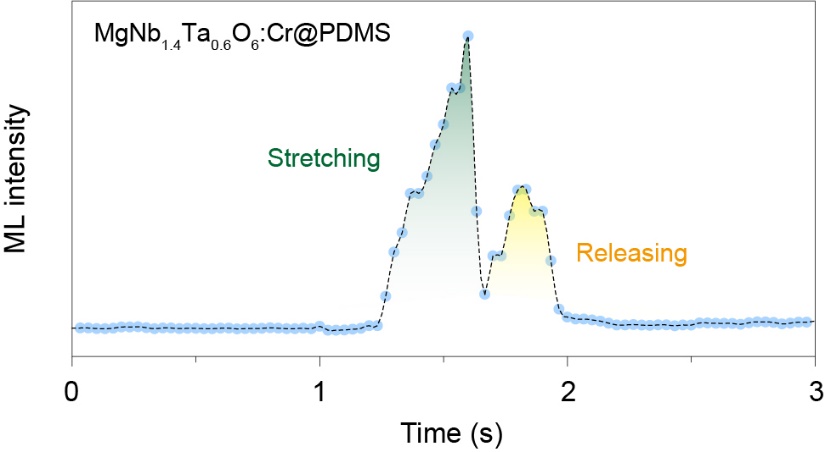
**

**Figure S25.** Time-resolved ML intensity curve of MgNb_1.4_Ta_0.6_O_6_:Cr^3+^@PDMS film upon a single stretching-releasing cycle at the speed and tensile strain of 30 mm/s and 20%, respectively. The observed double-peak profile revealed that ML occurs during both stretching and releasing, with the reduced ML intensity during releasing attributed to the weaker triboelectric effect in this stage.

**
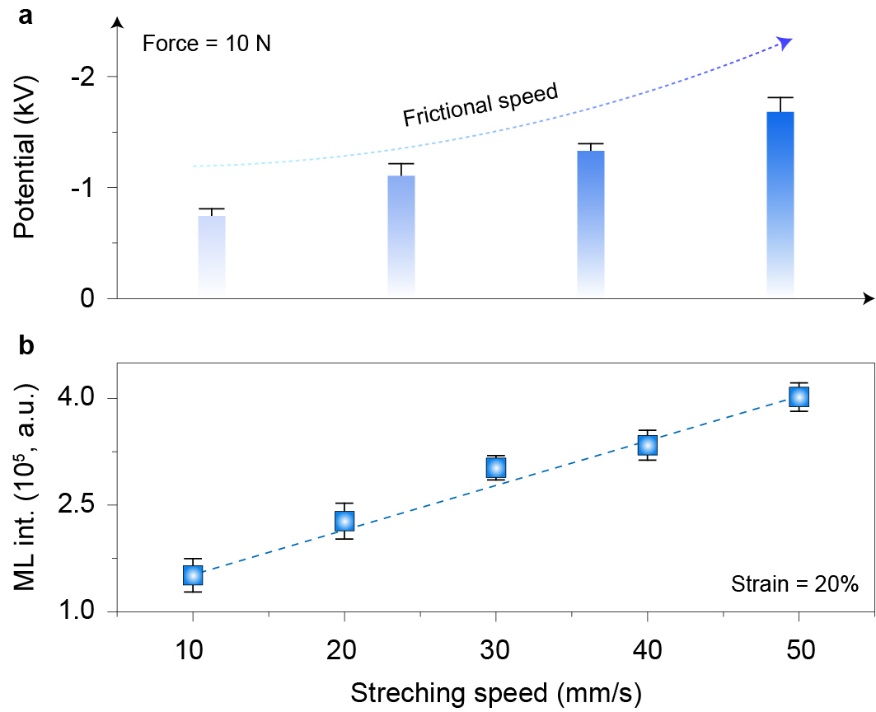
**

**Figure S26.** **a)** Triboelectric potential of the ML ceramics and PDMS film following 1 min of reciprocating friction under a load of 10 N at different speeds. **b)** The recorded ML intensity under different stretching speeds with a fixed tensile strain of 20%. Notably, the surface potential and ML intensity both gradually enhanced with the increase of the loading speed, further reinforcing the essential role of interfacial triboelectricity in the ML process.

**
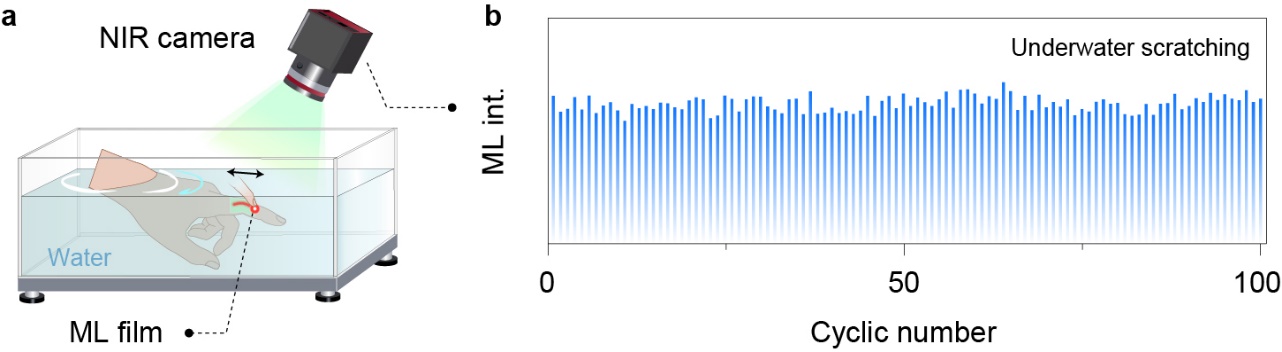
**

**Figure S27.** **a)** Schematic illustration of the experimental setup for underwater ML measurements under scratching modes. **b)** The cyclic stability of underwater ML intensity under repeated finger scratching actions. Note that the ML film was tightly adhered to the forefinger surface, and the ML signal during the finger movement was captured using a night-vision monocular positioned above the waterline. After immersion in aqueous solution, the composite elastomer largely preserved its highly reproducible and cyclically stable ML behavior under scratching actions.

**
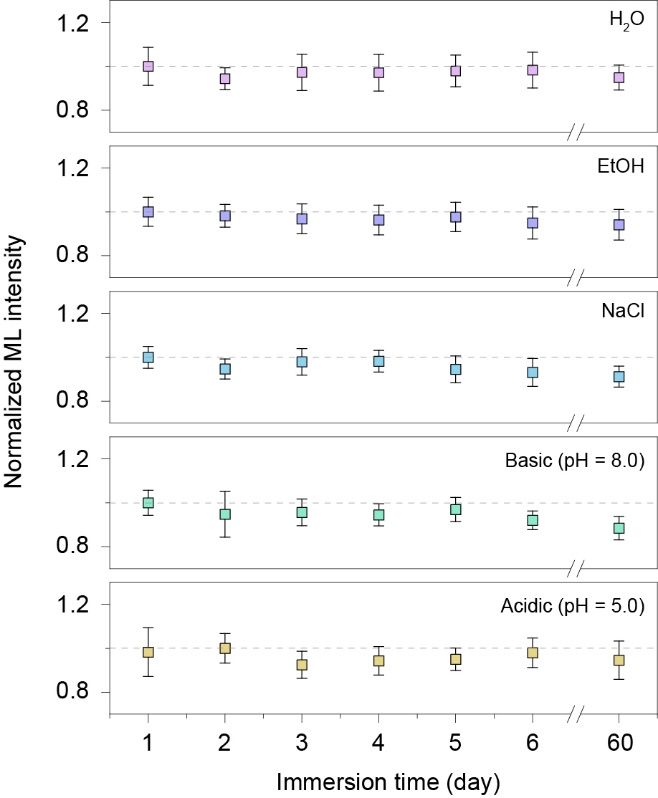
**

**Figure S28.** The ML stability of MgNb_1.4_Ta_0.6_O_6_:Cr^3+^@PDMS film after prolonged exposure to different chemical environments, including acidic aqueous solution (pH = 5), basic aqueous solution (pH = 8.0), concentrated NaCl solution (1 M), ethanol, and pure water. Note that the ML intensity was measured after the film was extracted from the solvent, dried, and subjected to friction excitation. It can be found that the SWIR-ML is highly resistant to a series of polar solvents even after prolonged soaking over two months. The slight ML degradation stems from the reduced triboelectric effect due to the inevitable water infiltration into microscopic voids of hydrophobic PDMS film.

**
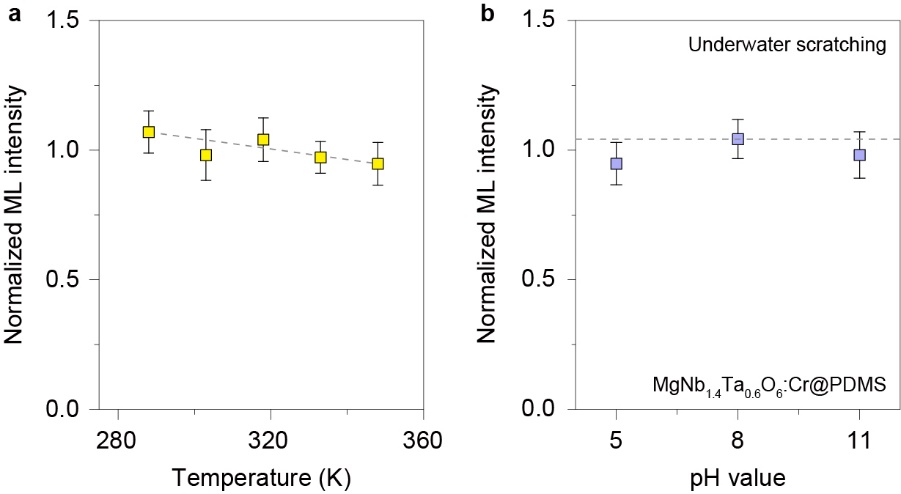
**

**Figure S29.** The ML variation of MgNb_1.4_Ta_0.6_O_6_:Cr^3+^@PDMS film as a function of **(a)** temperature (288, 303, 318, 333, 348 K) and **(b)** pH value (5, 8, 11) in water. Notably, the ML intensity showed a slight decrease under scratching as increasing the water temperature, due to the promotion of non-radiative relaxation (i.e., thermal quenching). Regarding ionic strength, higher concentrations of H^+^ and OH^-^ are present at pH = 5 and 11, respectively, where only a minor degradation of ML intensity was observed under these conditions. Although ionic species in water may partially dissipate triboelectric charges at exposed surfaces, the ML performance in present system is governed by the combined contribution of stress-induced piezoelectric effect and friction-induced triboelectric effect. The former is inherently insensitive to ionic strength, while the latter is mainly generated at the internal inorganic-organic interface rather than at the external force applicator-organic interface. Therefore, variations in ionic strength exerted only a limited influence on ML performance in the present material system.

**Table S1.** Comparison of self-recoverability and cyclic repeatability under different mechanical modes among the existing ML material systems.

| **Material** | **Type** | **ML range (nm)** | **Self-recoverability** | **Repeatability in powder**  **(*friction*)** | **Repeatability in ER**  **(*compressive*)** | **Repeatability in PDMS (*stretching*)** | **Ref.** |
| --- | --- | --- | --- | --- | --- | --- | --- |
| Sr_2_P_2_O_7_:Pr^3+^ | PersL-related | UV (230) | No | No | / | / | ^[14]^ |
| Ca_5_(PO_4_)_3_Cl:Eu | Triboelectric | Vis (465) | Yes | No | No | Yes **(> 1000)** | ^[4]^ |
| AlN | PersL-related | Vis (475) | No | No | / | / | ^[15]^ |
| CaBa_4_(PO_4_)_3_Cl:Eu | Triboelectric | Vis (498) | Yes | No | No | Yes **(> 1000)** | ^[16]^ |
| Ca_6_BaP_4_O_17_:Ce^3+^ | Triboelectric | Vis (488) | Yes | No | No | Yes **(~ 900)** | ^[17]^ |
| SrAl_2_O_4_:Eu^2+^/Dy^3+^ | PersL-related | Vis (520) | No | / | / | / | ^[18]^ |
| ZnS:Cu | Triboelectric | Vis (520) | Yes | / | / | Yes **(> 1000)** | ^[19]^ |
| Lu_3_Al_5_O_12_:Ce^3+^ | Triboelectric | Vis (530) | Yes | No | No | No **(**~ **5)** | ^[3]^ |
| CaF:Tb^3+^ | Triboelectric | Vis (544) | Yes | No | No | No **(~ 50)** | ^[20]^ |
| LiTaO_3_:Tb^3+^ | Piezoelectric | Vis (546) | Yes | / | Yes | / | ^[21]^ |
| Sr_2_SiO_4_:Eu^2+^ | PersL-related | Vis (570) | No | No | / | / | ^[22]^ |
| GaGa_4_O_7_:Mn^2+^ | PersL-related | Vis (579) | No | No | / | / | ^[23]^ |
| Li_0.5_Na_0.5_NbO_3_:Pr^3+^ | Piezoelectric | Vis (613) | Yes | / | Yes | / | ^[24]^ |
| Ca_3_Ga_4_O_9_:Eu^3+^ | Piezoelectric | Vis (617) | Yes | Yes | / | / | ^[25]^ |
| ZnS/CaZnOS:Mn^2+^ | Piezoelectric | Vis (620) | Yes | Yes | / | / | ^[8]^ |
| Y_3_Al_4_GaO_12_:Cr^3+^ | Piezoelectric | Vis (688) | Yes | / | **/** | No **(~ 10)** | ^[6]^ |
| Zn_3_Ga_2_GeO_8_:Cr^3+^ | PersL-related | Vis (700) | No | No | / | / | ^[26]^ |
| KGa_11_O_17_:Cr^3+^ | PersL-related | Vis (710) | No | No | / | / | ^[27]^ |
| LiGa_5_O_8_:Cr^3+^ | Piezoelectric | Vis (716) | Yes | Yes | / | / | ^[28]^ |
| LaGa_12_O_19_:Cr^3+^ | Piezoelectric | Vis (730) | Yes | Yes | Yes | / | ^[29]^ |
| Ga_2_O_3_:Cr^3+^ | Piezoelectric | Vis (740) | Yes | Yes | / | / | ^[5]^ |
| MgO/MgF:Cr^3+^ | Piezoelectric | NIR (820) | Yes | Yes | Yes | / | ^[30]^ |
| CaZnOS:Nd^3+^/Er^3+^ | Piezoelectric | SWIR (904) | Yes | Yes | Yes | / | ^[31]^ |
| SrZnOSe:Nd^3+^ | Piezoelectric | SWIR (918) | Yes | Yes | / | / | ^[32]^ |
| CaZnOS:Yb^3+^ | Piezoelectric | SWIR (980) | Yes | Yes | / | / | ^[33]^ |
| Ga_2_O_3_:Cr^3+^/Yb^3+^ | Piezoelectric | SWIR (1000) | Yes | Yes | / | / | ^[34]^ |
| Ba_2_ScSbO_6_:Cr^3+^ | Piezoelectric | SWIR (1010) | Yes | Yes | / | / | ^[7]^ |
| Sr_3_Sn_2_O_7_:Nd^3+^ | Piezoelectric | SWIR (1079) | Yes | / | Yes | / | ^[35]^ |
| **MgNb(Ta)_2_O_6_:Cr^3+^** | **Piezoelectric**  **Triboelectric** | **SWIR (960)** | **Yes** | **Yes** | **Yes** | **Yes (> 1000)** | **This work** |
| ^*^ “/” indicates that the corresponding data are not reported in the referenced work. | | | | | | | |

**Table S2.** Rietveld refinement and crystallographic results for MgNb_2_O_6_:Cr^3^⁺ crystals as a function of dopant concentration.

| **Cr^3+^ content** | **0%** | **0.5%** | **1%** | **2%** | **3%** | **4%** |  |
| --- | --- | --- | --- | --- | --- | --- | --- |
| **Space group** | *Pbcn* | | | | | | |
| ***Z*** | 6 | | | | | | |
| ***a* (Å)** | 14.190 | 14.188 | 14.188 | 14.188 | 14.186 | 14.183 |  |
| ***b* (Å)** | 5.7 | 5.7 | 5.7 | 5.7 | 5.7 | 5.69 |  |
| ***c* (Å)** | 5.03 | 5.03 | 5.03 | 5.03 | 5.03 | 5.03 |  |
| ***V*_[MgO6]_ (Å^3^)** | 14.3404 | 13.9342 | 13.6008 | 13.4539 | 13.3989 | 13.1831 |  |
| ***V*_[NbO6]_ (Å^3^)** | 9.5264 | 9.4858 | 9.1969 | 9.6954 | 9.3670 | 9.881 |  |
| ***V*_cell_ (Å^3^)** | 407.727 | 407.635 | 407.382 | 407.263 | 407.195 | 406.897 |  |
| ***R*_wp_** | 9.42% | 7.73% | 9.35% | 10.79% | 8.5% | 8.94% |  |
| ***R*_p_** | 7.16% | 6.06% | 7.3% | 7.94% | 6.6% | 6.99% |  |
| ***χ*^2^** | 2.61 | 3.6 | 2.61 | 3.41 | 2.03 | 2.34 |  |

**Table S3.** The calculated formation energy (*E_form_*) for MgNb_2_O_6_:Cr^3^⁺ crystal under different occupational situations.

| **Occupied sites** | ***E_T2_* (eV)** | ***E_pure_* (eV)** | ***E_Mg_* (eV)** | ***E_Nb_* (eV)** | ***E_Cr_* (eV)** | ***E_form_*(eV)** |
| --- | --- | --- | --- | --- | --- | --- |
| Cr → Mg (CN = 6) | -306.4303 | -306.4303 | -1.4748 | -10.2198 | -9.51056 | -4.6030 |
| Cr → Nb (CN = 6) | -309.8630 |  |  |  |  | -4.4546 |
| 2Cr → Nb & Mg (CN = 6) | -301.2664 |  |  |  |  | -4.3538 |
| *E_T2_* and *E_pure_* are the total energy of the doped and undoped crystals, respectively. | | | | | | |

**Reference**

[1] X. Zhang, H. Suo, Y. Guo, J. K. Chen, Y. Wang, X. H. Wei, W. L. Zheng, S. H. Li, F. Wang, *Nat. Commun.* **2024**, *15*, 6797.

[2] X. Zhang, H. Suo, Y. Z. Wang, B. Chen, W. L. Zheng, Q. K. Wang, Y. Wang, Z. X. Zeng, S.‐W. Tsang, D. Tu, F. Wang, *Laser Photonics Rev.* **2023**, *17*, 2300132.

[3] W. X. Wang, Z. B. Wang, J. C. Zhang, J. Y. Zhou, W. B. Dong, Y. H. Wang, *Nano Energy* **2022**, *94*, 106920.

[4] X. Pan, Y. X. Zhuang, W. He, C. J. Lin, L. F. Mei, C. J. Chen, H. Xue, Z. G. Sun, C. F. Wang, D. F. Peng, Y. Q. Zheng, C. F. Pan, L. X. Wang, R. J. *Nat. Commun*. **2024**, *15*, 2673.

[5] H. Suo, Y. Wang, X. Zhang, W. L. Zheng, Y. Guo, L. P. Li, P. L. Li, Y. M. Yang, Z. J. Wang, F. Wang, *Matter* **2023**, *6*, 2935.

[6] Z. C. Liu, X. Yu, Q. P. Peng, X. D. Zhu, J. Q. Xiao, J. T. Xu, S. X. Jiang, J. B. Qiu, X. H. Xu, *Adv. Funct. Mater*. **2023**, *33*, 2214497.

[7] C. Dou, T. L. Liang, M. Zhao, Z. Song, L. X. Ning, D. F. Peng, Q. L. Liu, *Adv. Funct. Mater*. **2024**, *34*, 2419716.

[8] D. F. Peng, Y. Jiang, B. L. Huang, Y. Y. Du, J. X. Zhao, X. Zhang, R. H. Ma, S. Golovynskyi, B. Chen, F. Wang, *Adv. Mater.* **2020**, *32*, 1907747.

[9] M. Ernzerhof, G. E. Scuseria, *J. Chem. Phys.* **1999**, *110*, 5029.

[10] J. Heyd, G. E. Scuseria, M. Ernzerhof, *J. Chem. Phys.* **2003**, *118*, 8207.

[11] C. X. Yuan, R. Y. Li, Y. F. Liu, L. L. Zhang, J. H. Zhang, G. Leniec, P. Sun, Z. H. Liu, Z. H. Luo, R. Dong, J. Jiang, *Laser Photonics Rev*. **2021**, *15*, 2100227.

[12] H. Dong, X. J. Zheng, W. Li, Y. Q. Gong, J. F. Peng, Z. Zhu, *J. Appl. Phys*. **2011**, *110*, 124109.

[13] C. J. Chen, Z. Lin, H. H. Huang, X. Pan, T.-L. Zhou, H. D. Luo, L. B. Jin, D. F. Peng, J. Xu, Y. X. Zhuang, R. J. Xie, *Adv. Funct. Mater.* **2023**, *33*, 2304917.

[14] H. M. Li, Y. M. Yang, P. Li, D. F. Peng, L. P. Li, *Adv. Mater*. **2024**, *36*, 2411804.

[15] T. Zheng, P. Woźny, K. Soler-Carracedo, D. X. Han, J. Wang, L. Peng, W. L. Li, D. F. Peng, H. L. Wu, J. Moszczyński, S. Mahlik, M. Runowski, *Adv. Mater*. **2025**, e11943.

[16] T. F. Fang, L. S. Zhao, X. He, B. Zhou, Y. W. He, Z. B. Lu, Z. F. Wang, *Adv. Mater*. **2025**, *37*, 2505071.

[17] S. Y. Qin, W. Y. Wei, B. R. Tian, Z. D. Ma, S. F. Fang, Y. S. Wang, J. C. Zhang, Z. F. Wang, *Adv. Funct. Mater*. **2024**, *34*, 2401535.

[18] Y. Deng, D. N. Peng, C. L. Shen, J. L. Sun, G. S. Zheng, S. L. Chang, Y. C. Liang, J. He, C. X. Shan, L. Dong, *Laser Photonics Rev*. **2024**, *18*, 2400251.

[19] H. I. Jeong, H. S. Jung, M. Dubajic, G. Kim, W. H. Jeong, H. Song, Y. Lee, S. Biswas, H. Kim, B. R. Lee, J. W. Yoon, S. D. Stranks, S. M. Jeong, J. Lee, H. Choi, *Nat. Commun*. **2025**, *16*, 854.

[20] W. X. Wang, S. W. Wang, Y. Gu, J. Y. Zhou, J. C. Zhang, *Nat. Commun*. **2024**, *15*, 2014.

[21] H. Yang, Y. Wei, H. N. Ju, X. R. Huang, J. Li, W. Wang, D. F. Peng, D. Tu, G. G. Li, Adv. *Mater*. **2024**, *36*, 2401296.

[22] Z. J. Ye, S. Q. Fang, T. C. Zhang, H. L. Cheng, J. Q. Ou, J. L. Yu, Y. X. Zhuang, R. J. Xie, L. Wang, *Adv. Mater*. **2025**, e14909.

[23] Y. Q. Tang, Y. Y. Cai, K. P. Dou, J. Q. Chang, W. Li, S. S. Wang, M. Z. Sun, B. L. Huang, X. F. Liu, J. R. Qiu, L. Zhou, M. M. Wu, J. C. Zhang, *Nat. Commun*. **2024**, *15*, 3209.

[24] X. X. Yang, R. Liu, X. H. Xu, Z. C. Liu, M. Z. Sun, W. Yan, D. F. Peng, C.-N. Xu, B. L. Huang, D. Tu, *Small*. **2021**, *17*, 2103441.

[25] L. W. Wu, J. W. Fan, H. X. Yao, T. Wei, J. Li, J. W. Yan, T. Y. Wang, C. L. Zhou, Y. D. Han, L. Y. Li, D. Tu, D. L. Geng, *Adv. Funct. Mater*. **2025**, e16475.

[26] Q. Liu, Y. T. Zheng, D. F. Peng, J. Zhao, Z. Song, Q. L. Liu, *Adv. Funct. Mater*. **2023**, *33*, 2209275.

[27] C. Dou, J. G. Gao, Z. Song, L. X. Ning, Q. L. Liu, *Adv. Funct. Mater*. **2025**, *35*, 2508216.

[28] P. X. Xiong, B. L. Huang, D. F. Peng, B. Viana, M. Y. Peng, Z. J. Ma, *Adv. Funct. Mater*. **2021**, *31*, 2010685.

[29] X. S. Wang, Y. Xiao, P. X. Xiong, P. Zheng, S. B. Xu, W. Z. He, H. Q. Fang, P. Z. Wang, J. Y. Yu, S. Wu, Q. Qian, *Adv. Funct. Mater*. **2025**, e15717.

[30] S. Wu, S. Y. Wang, Z. G. Shao, Y. Z. Wang, P. X. Xiong, *Nat. Commun*. **2025**, *16*, 8912.

[31] P. Zheng, Y. Xiao, P. X. Xiong, S. J. Su, A. P. Yang, X. S. Wang, S. B. Xu, P. S. Shao, Z. Y. Zhou, S. Wu, E. H. Song, J. L. Gan, D. D. Chen, *Adv. Funct. Mater*. **2025**, *37*, 2505094.

[32] Y. Wang, B. Y. Ren, W. L. Zheng, D. F. Peng, F. Wang, *Adv. Mater*. **2024**, *36*, 2406899.

[33] Y. Y. Du, Y. Jiang, T. Y. Sun, J. X. Zhao, B. L. Huang, D. F. Peng, F. Wang, *Adv. Mater*. **2019**, *31*, 1807062.

[34] S. Q. Liu, Y. Guo, Z. Song, D. F. Peng, Q. L. Liu, F. Wang, *Adv. Mater*. **2025**, *37*, e06957.

[35] T. Dong, C. N. Xu, S. Kamimura, Y. Horibe, H. Oshiro, L. Zhang, Y. Ishii, K. Hyodo, G. Marriott, N. Ueno, X. G. Zheng, *Adv.*
